# Environmental Bacteria Involved in Manganese(II) Oxidation and Removal From Groundwater

**DOI:** 10.3389/fmicb.2019.00119

**Published:** 2019-02-11

**Authors:** Ainelén Piazza, Lucila Ciancio Casalini, Virginia A. Pacini, Graciela Sanguinetti, Jorgelina Ottado, Natalia Gottig

**Affiliations:** ^1^Instituto de Biología Molecular y Celular de Rosario (IBR), Consejo Nacional de Investigaciones Científicas y Técnicas (CONICET) y Universidad Nacional de Rosario (UNR), Rosario, Argentina; ^2^Centro de Ingeniería Sanitaria, Universidad Nacional de Rosario, Rosario, Argentina

**Keywords:** manganese-oxidizing bacteria, manganese removal, biofilm, groundwater, Argentina

## Abstract

The presence of iron (Fe) and manganese (Mn) in groundwater is an important concern in populations that use it as source of drinking water. The ingestion of high concentrations of these metals may affect human health. In addition, these metals cause aesthetic and organoleptic problems that affect water quality and also induce corrosion in distribution networks, generating operational and system maintenance problems. Biological sand filter systems are widely used to remove Fe and Mn from groundwater since they are a cost-effective technology and minimize the use of chemical oxidants. In this work, the bacterial communities of two biological water treatment plants from Argentina, exposed to long term presence of Mn(II) and with a high Mn(II) removal efficiency, were characterized using 16S rRNA gene Illumina sequencing. Several selective media were used to culture Mn-oxidizing bacteria (MOB) and a large number of known MOB and several isolates that have never been reported before as MOB were cultivated. These bacteria were characterized to select those with the highest Mn(II) oxidation and biofilm formation capacities and also those that can oxidize Mn(II) at different environmental growth conditions. In addition, studies were performed to determine if the selected MOB were able to oxidize Mn(II) present in groundwater while immobilized on sand. This work allowed the isolation of several bacterial strains adequate to develop an inoculum applicable to improve Mn(II) removal efficiency of sand filter water treatment plants.

## Introduction

In Argentina, groundwater provides drinking water to a large part of the population, and the presence of iron (Fe) and manganese (Mn) in these waters is a common concern (Pacini et al., 2005). Water supplies containing Fe and Mn concentrations exceeding the permitted limits may produce potential undesirable effects on human health. The ingestion of high concentrations of these metals may cause mild symptoms such as anorexia, weakness, apathy and learning and/or understanding problems, but can also cause serious diseases such as Parkinson’s or Alzheimer’s disease (Avila et al., 2013; Farina et al., 2013; Bjorklund et al., 2017). In addition, these metals cause aesthetic, organoleptic and operating problems affecting the quality of finished water (Sly et al., 1990) and can induce corrosion in water distribution systems (Pacini et al., 2005). Therefore, purification of groundwater containing these metals plays an important role in environmental and social safety.

In groundwater, Fe and Mn are present mainly as reduced soluble forms Fe(II) and Mn(II), and removal processes are based on the induction of oxidation of these metals to form insoluble oxides that can be filtered out of the water. This may be achieved by physico-chemical methods or by a more efficient and eco-friendly strategy which involves biological treatments (Mouchet, 1992; Vandenabeele et al., 1992; Katsoyiannis and Zouboulis, 2004; Burger et al., 2008a; Tekerlekopoulou et al., 2008). In Argentina, biological sand filter technology is widely used for potabilization of groundwater (Pacini et al., 2005). In this process, Fe(II) is easily oxidized by aeration and retained in the filtration system, therefore this metal is quickly removed with high efficiency (Pacini et al., 2005, 2014). The oxidation of Mn(II) to form Mn(III/IV) oxides by O_2_ is a kinetically limited process and this metal cannot be effectively removed by simple aeration and precipitation (Diem and Stumm, 1984; Pacini et al., 2014). MOB can increase the rate of Mn(II) oxidation by up to 5 orders (Katsoyiannis and Zouboulis, 2004; Bai et al., 2013). Therefore, to improve Mn(II) removal processes, sand filters may be inoculated with suitable bacteria that can oxidize Mn(II) and form biofilms that precipitate Mn oxides on these filters (Bai et al., 2016; Li et al., 2016).

Manganese-oxidizing bacteria play a central role in the biogeochemical cycle of Mn, having a deep impact on the Earth’s biogeochemistry (Tebo et al., 2005; Hansel and Learman, 2016). They belong to several bacterial phyla including Firmicutes, Actinobacteria, Bacteroidetes, and Proteobacteria. These bacteria are ubiquitous in nature and can be isolated from different habitats. Furthermore, there is evidence that those habitats containing high levels of Mn and those in which Mn cycling is an active process tend to have high numbers of MOB (Tebo et al., 2005; Hansel and Learman, 2016). Several studies have been performed to understand the mechanisms involved in Mn oxide formation through different phyla. In most bacteria, Mn(II) oxidation starts in stationary phase and Mn(II) oxidase activity is localized to the outer surface of bacterial cells, therefore, Mn oxides cover these cells (Tebo et al., 2005; Hansel and Learman, 2016). Enzymes involved in Mn(II) oxidation were identified in some bacteria and include multicopper oxidases and peroxidases (Geszvain et al., 2012). Some studies demonstrated that MOB could derive energy from Mn(II) oxidation (Ehrich and Salerno, 1990) and also that this process enhances resistance to the reactive oxygen species hydrogen peroxide (Banh et al., 2013). However, the physiological function of bacterial Mn(II) oxidation remains unclear (Tebo et al., 2005).

The aim of this work was to find novel and autochthonous culturable MOB to develop a bacterial inoculum applicable to accelerate and enhance Mn removal in groundwater treatment plants. Therefore, the microbiomes of two groundwater biological treatment plants that currently remove Mn(II) with high efficiency, one located in las toscas (LT) and the other one in villa ocampo (VO), were evaluated. In both plants, the treatment line comprises a stage of aeration, followed by two filtration steps: one in a roughing pre-filter (PF) composed of a gravel bed and the other ones in a conventional rapid filter of sand bed (Pacini et al., 2014). A large number of known bacterial genera capable of Mn(II) oxidation and several isolates that have never been reported before to have this activity were cultivated. Furthermore, selection of the best MOB was performed through the study of their Mn(II) oxidation and biofilm formation capacities and by analyzing their capacity to oxidize Mn(II) adaptively in a changing environment and in groundwater with these bacteria immobilized on sand. Overall, these studies allowed the selection of potentially outstanding bacterial candidates suitable for future inoculations to improve groundwater Mn(II) removal processes.

## Materials and Methods

### Sample Collection and Physico-Chemical Characteristics of Groundwater and Effluent Treated Water

Samples were collected from two full-scale biological filtration plants located in the municipalities of VO (28°29′20.8″S 59°20′29.5″W) and LT (28°21′28.2″S 59°15′48.2″W), in the province of Santa Fe, Argentina. VO plant has been in service since 2011 and LT plant since 2013 and both systems were built using the same technology. Typical values of the dominant chemical constituents in groundwater and performance of the treatment are listed in (Supplementary Table S1). Groundwater samples were collected from the main tank influent under anoxic conditions before the aeration unit. Groundwater and effluent treated water quality data were quantified at Centro de Ingeniería Sanitaria, Universidad Nacional de Rosario using standard methods (Rice et al., 2017). The filtering material, gravel (2030 mm size) from the PFs and sand (1.10–2.20 mm size) from the filters, were hand collected in sterile polyethylene bottles. Collected materials used to cultivate and isolate bacteria were immediately put on ice and then preserved at 4°C until use. Part of the material was used for DNA extractions; and in this case samples were immediately frozen in liquid N_2_ and kept frozen until use.

### DNA Extraction, 16S rRNA Gene Library Preparation, Sequencing and Taxonomic Assignment

Genomic microbial DNA was extracted from sand samples using PowerMax^®^ Soil DNA Isolation Kit (MO BIO Laboratories, Inc.), following the manufacturer’s instructions. The amount of DNA was quantified using Quant-iT^TM^ PicoGreen^®^ dsDNA assay kit (Invitrogen Corporation, Carlsbad, CA, United States). 16S rRNA gene library was made based on the procedure described in Sample Preparation Guide–Illumina. Briefly, for library preparation, the V3 and V4 region of the 16S rRNA gene was amplified from 12.5 ng of DNA. This 16S rRNA gene region was amplified by polymerase chain reaction (PCR) (95°C for 3 min, followed by 25 cycles at 95°C for 30 s, 55°C for 30 s and 72°C for 30 s, followed by a final extension at 72°C for 5 min) using the primers: 341F: 5′-TCGTCGGCAGCGTCAGATGTGTATAAGAGACAGCCTACGGGNGGCWGCAG-3′ and 805R: 5′-GTCTCGTGGGCTCGGAGATGTGTATAAGAGACAGGACTACHVGGGTATCTAATCC-3′, in which Illumina overhang adapter sequences added to locus-specific sequences at the 5′ are underlined. PCRs were performed in triplicate, meaning each sample was amplified in 3 replicate 25-μL PCR reactions containing 2.5 μL of DNA (5 ng/μl), 5 μL of each primer (1 μM) and 12.5 μL of 2x KAPA HiFi HotStart ReadyMix. Triplicate PCR reactions for each sample were pooled into a single volume (75 μL) and purified using the AMPure XP beads (Beckman Coulter Life Sciences, Indianapolis, IN, United States) according to the manufacturer’s instructions. Then Index PCR (95°C for 3 min, followed by 8 cycles at 95°C for 30 s, 55°C for 30 s and 72°C for 30 s, followed by a final extension at 72°C for 5 min) using the Nextera XT Index Primers (N7xx and S5XX) were performed in 50-μL mixture containing 5 μL of DNA, 5 μL of each primer (1 μM), 25 μL of 2x KAPA HiFi HotStart ReadyMix and 10 μL of PCR Grade water. A second purification with AMPure XP beads (Beckman Coulter Life Sciences, Indianapolis, IN, United States) was performed to clean up the final library. The library was quantified using Quant-iT^TM^ PicoGreen^®^ dsDNA assay kit (Invitrogen Corporation, Carlsbad, CA, United States) and denatured with NaOH before sequencing on Illumina MiSeq platform (Illumina, Inc.).

Sequence data pre-processing, classification and taxonomic assignment was done as previously described (Piñero et al., 2017). Briefly, quality control of the reads was made using FastQC v0.11.5 5^1^ and multiQC^2^. The joining of the reads was done using the software PEAR^3^. Filtering of the reads by quality and length was done with FASTQX-Toolkit programs^4^ and BBMap^5^. Concatenated and filtered FASTQ sequences were converted to FASTA format and sequences that contain ‘N’ were removed. To remove the sequences identified as chimeras, VSEARCH software was used^6^ taking as reference the database of the Ribosome Database Project (RDP^7^). The remaining sequences were clustered to operational taxonomic units (OTUs) at 97% similarity level with an open reference strategy implemented in QIIME^8^, using SortMeRNA^9^ for the reference picking against SILVA (v119) database and SUMACLUST^10^ for *de novo* OTU picking. Taxonomic classification was implemented using the assigned taxonomy by script included in QIIME. This was performed by the facility of the Instituto de Agrobiotecnología Rosario^11^. Illumina sequences were deposited in SRA database which is available at NCBI with SRA accession: PRJNA514808.

### Selection and Isolation of MOB

Representative portions of gravel from PFs and sand from filters (5 g) were taken and suspended aseptically in 50 mL sterile 0.85% (w/v) NaCl isotonic solution and vortexed vigorously to disaggregate biofilms. Appropriate dilutions of these suspensions were prepared (10^-1^ to 10^-5^) and 0.1 mL from each dilution were plated on three different culture media: PC-Medium (0.05 g/L yeast extract) (Tyler and Marshall, 1967), Mn-Medium [0.001 g/L FeSO_4_.7H_2_O, 2.0 g/L Peptone, 0.5 g/L yeast extract, 10 mM HEPES buffer (*N*-2-hydroxyethylpiperazine-*N*′-2-ethanesulfonic acid, pH 7.5)] (Stein et al., 2001) and Lept-medium (0.5 g/L yeast extract, 0.5 g/L casamino acids, 5 mM glucose, 0.48 mM CaCl_2_, 0.83 mM MgSO_4_, 3.7 μM FeSO_4_.7H_2_O, 10 mM HEPES pH 7.5, 0.15 μM ZnSO_4_, 0.08 μM CoCl_2_, 0.06 μM Na_2_MoO_4_) (Boogerd and de Vrind, 1987). Culture media for VO samples were prepared using VO groundwater and media for LT samples were prepared with LT groundwater, instead of distilled water. VO groundwater contain 8 μM Mn (∼0.43 mg/L Mn) and LT 6 μM Mn (∼0.33 mg/L Mn) (Supplementary Table S1), and MnSO_4_ was supplemented to reach a 100 μM final concentration. The plates were incubated at 28°C and examined every day for the development of colonies with dark brown centers or edges as evidence of Mn oxides depositions. The identified dark colonies were then isolated and plated again on the same media with or without 100 μM MnSO_4_, to confirm that in the absence of added Mn(II) the colonies of MOB remained whitish. The colonies were also stained with Leucoberbelin Blue (LBB) dye solution (0.04% w/v LBB in 45 mM acetic acid) to corroborate that brown color corresponded to Mn oxides. In the presence of Mn(III) or Mn(IV), the LBB dye is oxidized producing a blue color (Krumbein and Altmann, 1973; Tebo et al., 2007) (Supplementary Figure S1). A total of 202 Mn-oxidizing colonies were obtained, picked and streaked for isolation. Cultures of these MOB grown in LB medium (Bertani, 1951) were supplemented with 20% glycerol and frozen (-70°C) for storage.

### Molecular Identification of Cultured MOB and Phylogenetics Studies

Single colonies of the 202 cultured MOB were suspended in 30 μL of sterile distilled water and heated at 94°C for 8 min. Aliquots of 5 μL were taken as template to carry out 16S rRNA gene PCR amplification using the primers pair FD1 and RP1 (Clarridge, 2004). The amplification products (1500 bp) were purified with Wizard^®^ SV Gel and PCR Clean-Up System (Promega) and were sequenced by Sanger Sequencing at the University of Maine. This facility uses the BigDye^TM^ Terminator v3.1 Cycle Sequencing Kit, an automated version of Sanger sequencing, and the sequencing reactions were run on an ABI (Applied Biosystems) 3730 Sequencer using a 50 cm capillary array. Both 16S rRNA gene strands were sequenced and reverse sequences were transformed in reverse complement sequences using Seq-Builder module of Lasergene 7 software package DNASTAR (Burland, 2000). These sequences were aligned with forward sequences using MegAlign module of the same package (Burland, 2000). The obtained consensus nucleotide sequences were compared to 16S rRNA gene sequences deposited at SILVA database to determine bacteria identities.

Alignments of the 16S rRNA sequenced genes were made in order to group all the isolates that showed unique sequences and single representatives of each group were chosen for further studies. Phylogenetic trees based on 16S rRNA gene sequences were constructed by maximum-likelihood method (Felsenstein, 1981) and the Kimura 2 parameter model using MEGA 5 (Tamura et al., 2007). The topology of phylogenetic trees was evaluated by bootstrap method with 100 replications. *Escherichia coli* 16S rRNA gene was used as the out-group and they are shown in Supplementary Figure S2 for isolates with the best Mn(II) oxidation performance. The 16S rRNA gene sequences of the 23 MOB characterized in this work are publicly available at GenBank (accession numbers MK011855 to MK011877, from MOB-104 to MOB-382, as shown in Table 1). These sequences were searched in the Illumina sequencing data and the proportion of reads with more than 97% similarity between both datasets were quantified as previously described (Rascovan et al., 2016).

**Table 1:** Identification of closest related species of the 23 strains studied in this work and description of their localization at LT pre-filter, LT filter, VO pre-filter and VO filter.

Name (GenBank accession number)	Plant	Selection medium	Closest related species (GenBank accession numbers – % of similarity)
MOB-104 (MK011855)	VO pre-filter	PC	*Pseudomonas resinovorans* (KP322753.1 - 97%)
			*Pseudomonas resinovorans*^a^
MOB-109 (MK011856)	VO pre-filter	PC	*Noviherbaspirillum malthae* (NR_115809.1 - 97%)
MOB-111 (MK011857)	VO pre-filter	PC	*Noviherbaspirillum malthae* (NR_115809.1 - 96%)
MOB-206 (MK011858)	LT filter	PC	*Pseudomonas resinovorans* (AP013068.1 - 99%)
			*Pseudomonas alcaligenes* (MG905251.1 - 99%)
			*Pseudomonas* sp. (KM187334.1 - 99%)
MOB-228 (MK011859)	LT filter	PC	*Pseudomonas jinjuensis* (KF815696.1 - 98%)
			*Pseudomonas citronellolis* (JF911368.1 - 98%)
			*Pseudomonas* sp. (DQ118950.1 - 98%)
MOB-243 (MK011860)	LT filter	PC	*Pseudomonas* sp. (EU043328.1 - 99%)
			*Pseudomonas* sp. (EU043318.1 - 99%)
			*Pseudomonas* sp. (EU043320.1 - 99%)
			*Pseudomonas flavescens*^a^
MOB-244 (MK011861)	LT filter	PC	*Pseudomonas resinovorans* (MG011582.1 - 97%)
			*Pseudomonas resinovorans*^a^
MOB-342 (MK011862)	VO pre-filter	PC	*Pseudomonas* sp. (MH337930.1 - 98%)
			*Pseudomonas* sp. (HF545851.1 - 98%)
			*Pseudomonas chlororaphis*^a^
MOB-343 (MK011863)	VO pre-filter	PC	*Pseudomonas resinovorans* (MF943158.1 - 99%)
			*Pseudomonas alcaligenes* (KM272279.1 - 98%)
MOB-364 (MK011864)	LT filter	PC	*Pseudomonas* sp. (AB047273.1 - 97%)
			*Pseudomonas resinovorans* (AP013068.1 - 97%)
MOB-436 (MK011865)	VO pre-filter	PC	*Pseudomonas resinovorans* (KJ870047.1 - 99%)
			*Pseudomonas alcaligenes* (MF682034.1 - 99%)
			*Pseudomonas pseudoalcaligenes* (KJ586277.1 - 99%)
			*Pseudomonas linyingensis* (NR_117838.1 - 98%)
MOB-180 (MK011866)	LT filter	Lept	*Pseudomonas* sp. (AF326380.1 - 98%)
			*Pseudomonas* sp. (FJ379321.1 - 98%)
MOB-181 (MK011867)	LT filter	Lept	*Pseudomonas guangdongensis* (NR_118458.1 - 97%)
			*Pseudomonas linyingensis* (NR_117838.1 - 97%)
			*Pseudomonas sagittaria* (NR_118347.1 - 96%)
MOB-182 (MK011868)	LT filter	Lept	*Pseudomonas* sp. (AF326380.1 - 99%)
			*Pseudomonas* sp. (FJ379321.1 - 99%)
			*Pseudomonas* sp. (JX416377.1 - 99%)
MOB-257 (MK011869)	LT filter	Lept	*Flavobacterium* sp. (KF499502.1 - 98%)
			*Flavobacterium* sp. (DQ205296.1 - 98%)
MOB-326 (MK011870)	VO filter	Lept	*Terrabacter tumescens* (NR_044984.2 - 98%)
MOB-412 (MK011871)	LT filter	Lept	*Pseudomonas resinovorans*^a^
MOB-449 (MK011872)	VO filter	Lept	*Pseudomonas resinovorans* (KY368643.1 - 96%)
			*Pseudomonas alcaligenes* (KM272279.1 - 96%)
			*Pseudomonas resinovorans*^a^
MOB-505 (MK011873)	LT filter	Lept	*Pseudomonas resinovorans* (MG011582.1 - 98%)
			*Pseudomonas resinovorans*^a^
MOB-513 (MK011874)	LT filter	Lept	*Pseudomonas* sp. (KC782842.1 - 96%)
			*Pseudomonas* sp. (FJ959376.1 - 96%)
			*Pseudomonas* sp. (MG266358.1 - 96%)
MOB-68 (MK011875)	VO pre-filter	Mn	*Ensifer* sp. (MF621571.1 - 99%)
			*Ensifer adhaerens* (EU095366.1 - 99%)
MOB-58 (MK011876)	VO pre-filter	Mn	*Rhizobium* sp. (KX611611.1 - 99%)
			*Rhizobium* sp. (KY575353.1 - 99%)
MOB-382 (MK011877)	VO filter	Mn	*Sphingomonas leidyi*^a^


*Bacterial species were identified by construction of phylogenetic trees based on 16S rRNA gene sequences and MALDI-TOF MS analysis. ^*a*^Indicates that these bacterial were identified at species level by MALDI-TOF (with score values ≥ 2.0).*

### Biotyper MALDI-TOF MS Analysis

Bacterial identification was carried out directly using Matrix-assisted laser desorption ionization–time of flight mass spectrometry (MALDI-TOF MS) (Welker and Moore, 2011; Dingle and Butler-Wu, 2013). Single colonies of freshly grown bacteria were transferred in duplicate from a culture plate directly onto a target plate using sterile toothpicks. The spotted bacteria were then overlaid onto a MALDI target plate with 1 μL of 70% formic acid to lyse bacteria. Immediately 0.5 μl of the MALDI matrix solution (70/30 acetonitrile/water) was added. Once the matrix and lysed bacteria spots were dry, the target plate was loaded into the ionization chamber of the MALDI-TOF MS instrument (Bruker Daltonics, Bremen, Germany) where samples were ionized. The ionized molecules are accelerated by an electric charge and travel through a vacuum tube toward a detector. As they travel, the ionized molecules are separated based on their mass-to-charge ratio (i.e., m/z). The mass analyzer of the instrument precisely measures the time required for each ion to reach the detector and records the TOF, generating a mass spectrum for each bacteria. FlexControl software (version 3.4) was used for measurements. The spectra were analyzed using BioTyper software (version 3.1.66; Bruker) and database MBT DB-5627 provided by the manufacturer. The MALDI-TOF MS identification scores for the species were ≥2.0.

### Mn(II) Oxidation Assays

Taking into account that the selected strains will be used to inoculate water treatment plants where they will have to oxidize Mn(II) attached to the sand filters, quantification of Mn(II) oxidation was performed on solid media, using LBB dye, as previously described (Krumbein and Altmann, 1973; Tebo et al., 2007), with the following modifications. Bacteria were grown in LB with shaking until exponential growth phase and diluted to an absorbance at 600 nm (OD_600_) = 0.1 in Mn oxidation culture media where the bacteria were selected (Table 1). 20 μL drops of these dilutions (OD_600_ = 0.1) were spotted on these media containing MnSO_4_ 100 μM. Plates without Mn(II) in the culture medium were the controls. At different time-points (days) cells were removed from drops with a loop and resuspended in 1 mL of 10 mM buffer HEPES (pH = 7.5). 500 μL of 0.04% (w/v) LBB solution were added to quantify Mn oxides produced. The samples were vortexed for 30 s and centrifuged to remove cells. Thereafter, the supernatants absorbance was measured at 618 nm using a Synergy 2 Reader, BioTek. To standardize the measurements among bacteria, total protein was quantified. Each cell pellet was washed once with 10 mM HEPES buffer (pH = 7.5), resuspended in 1 mL of the same buffer, sonicated on ice with a 3 mm probe with six cycles of 10 s on and 1 min off with 25% amplitude (Sonics Materials^TM^ VC 750 Ultrasonic Processor); and total proteins quantified by Bradford method (Bradford, 1976). The capacity of Mn(II) oxidation was calculated as absorbance at 618 nm/total protein concentration (mg/mL). Four independent experiments were performed and the mean values and standard deviation (SD) are shown in the figures. Data were statistically analyzed using one-way analysis of variance (ANOVA) (*P* < 0.05). These assays were also performed at different growth temperatures (18, 28, and 37°C) and varying the initial Mn(II) concentrations (MnSO_4_ 100, 150, and 200 μM) and in the presence of Fe(II) (FeSO_4_ 5, 10, 25, and 100 μM).

### Bacterial Growth

Bacteria were grown as described above on appropriate medium containing 100 μM MnSO_4_. Plates without Mn(II) in the culture medium were the controls. At different time-points (days) cells were removed from drops with a loop and resuspended in 1 mL of 0.85% (w/v) NaCl, followed by serial dilutions, and plating onto PC agar plates. Colonies were counted after 48 h of incubation at 28°C, and the results are presented as log CFU/mL. All quantifications were performed at least in triplicate for both with and without Mn(II) conditions and the mean values and standard deviation (SD) are shown in the figures. Data were statistically analyzed using one-way analysis of variance (ANOVA) (*P* < 0.05).

### Biofilm Assays

For biofilm formation assays, overnight bacterial cultures were diluted 1:10 and grown in LB medium with shaking until exponential growth phase and to the same bacterial concentration and then diluted 1:10 in fresh LB medium. A total of 2 mL of diluted bacterial suspension was placed in LB medium with or without 100 μM MnCl_2_ in borosilicate glass tubes and incubated statically at 28°C. At different time-points, biofilms were quantified by Crystal Violet (CV) staining as previously described (Zimaro et al., 2014). All assays were performed at least in triplicate for both with and without Mn conditions. Values represent the mean from three tubes for each strain; data were statistically analyzed using ANOVA (*P* < 0.05).

### MOB Growth Interaction Assays

The growth interactions among strains were studied as previously described (Li et al., 2016). Briefly, 100 μL of an overnight culture of each strain were spread onto LB agar. After drying, drops of an overnight culture of each strain were placed onto the surfaces of the LB agar plates, and incubated at 28°C for 24–48 h. The growth inhibition of one strain by another was measured by determining the diameter of the inhibition zone (halo) surrounding the drops.

### Quantification of Groundwater Mn(II) Oxidation Performed by Sand Immobilized MOB

Bacteria grown until stationary phase in LB medium were harvested by centrifugation and resuspended in LB at 1 g of fresh bacterial pellet per L of LB. 50 mL of these bacterial suspensions were incubated with 80 g of sterilized sand (1.10–2.20 mm effective size) in 1 L Erlenmeyer flasks for 5 days. Sands not inoculated with bacteria were used as controls. Then, LB medium was removed, sand was washed four times with sterile water and incubated at 30°C with 50 mL of groundwater from VO (Supplementary Table S1) supplemented with MnCl_2_ to reach 100 μM final concentration. After 7 days of incubation 1 mL of each groundwater supernatant was mixed with 500 μL of 0.04% (w/v) LBB solution in 45 mM acetic acid and absorbance was measured at 618 nm using a Synergy 2 Reader, BioTek. The concentration of Mn oxides was determined using calibration curves that were generated with Mn(IV) in a linear range from 0 to 150 μM. Three independent experiments were performed. Data were statistically analyzed using ANOVA (*P* < 0.05).

## Results and Discussion

### Physico-Chemical Parameters of Groundwater From Las Toscas and Villa Ocampo

Groundwater and effluent water samples were collected from LT and VO treatment plants and physico-chemical parameters were analyzed. The main differences between both groundwater qualities were higher concentrations of sulfate, fluoride and sodium in LT than in VO. Currently, both plants can remove Mn(II) and Fe(II) with high efficiency (Supplementary Table S1) and effluent water quality parameters are within the acceptable limits established by Argentine law (Law No. 11,220 Annex A). Due to these high Mn removal efficiencies, both plants were chosen to investigate their bacterial communities and to isolate culturable MOB.

### Characterization of Bacterial Diversity Associated to Las Toscas and Villa Ocampo Biofilters

Since in biological treatment plants Mn(II) removal is taking place in both PF and filter (Pacini et al., 2005), samples were collected from all these filtration stages in LT and VO treatment plants. Bacterial communities of these samples were characterized using 16S rRNA gene Illumina sequencing. LT PF, LT filter and VO PF had the highest species richness, and VO filter samples had the lowest species richness of all the bacterial communities (Supplementary Figure S3A). The bacterial diversity, estimated by Shannon diversity index from OTU data, was highest for LT filter samples followed by LT PF and VO PF and the lowest value was observed in VO filter (Supplementary Figure S3B).

**FIGURE 1:**
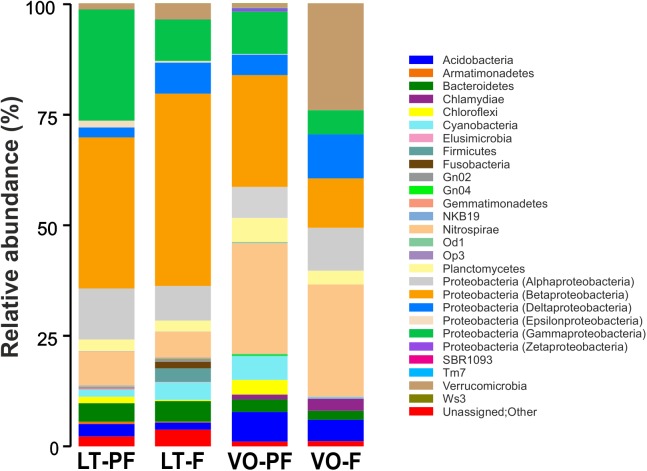
Taxonomic composition of bacteria from LT pre-filter (LT-PF), LT filter (LT-F), VO pre-filter (VO-PF) and VO filter (VO-F) samples. Relative taxa abundance are represented along the vertical axis with different colors.

Sequence analysis of OTUs indicated that either in LT and VO, the dominant phyla were Proteobacteria with a majority of Betaproteobacteria, Gammaproteobacteria, Alphaproteobacteria and Deltaproteobacteria subclasses (Figure 1 and Supplementary Table S2). In both LT and VO Nitrospirae was the second most abundant phylum and also Acidobacteria, Firmicutes, Chloroflexi, Planctomycetes, Cyanobacteria, and Bacteroidetes were found. Verrucomicrobia and Chlamydiae phylum were abundant in VO filter samples (Figure 1 and Supplementary Table S2). Consistent with these results, an important role of Proteobacteria for Mn removal in sand filter systems had been demonstrated (Nitzsche et al., 2015; Gulay et al., 2016). In addition, several OTUs from LT and VO corresponded to well-known MOB (Supplementary Table S3): *Pseudomonas* (Cerrato et al., 2010; Geszvain et al., 2016), *Crenothrix* (Cheng et al., 2017), *Hydrogenophaga* (Marcus et al., 2017), *Leptothrix* (Hope and Bott, 2004; Burger et al., 2008b), *Erythrobacter* (Francis et al., 2001), *Hyphomicrobium* (Northup et al., 2003), *Variovorax* (Stein et al., 2001), *Rhodobacter* (Templeton et al., 2005), *Sphingomonas* (Francis et al., 2001), *Flavobacterium* (Akob et al., 2014) and *Acidovorax* (Tsuji et al., 2017). Also, known iron-oxidizing bacteria like *Gallionella* and *Rheinheimera* (Chen et al., 2013; Fleming et al., 2014; Yang et al., 2014) were present in both LT and VO consistent with the presence of Fe(II) in both groundwaters (Supplementary Table S2). Interestingly, Verrucomicrobia phylum was abundant in VO filter suggesting a role of these bacteria in Mn removal, however, these bacteria have never been related to groundwater biological treatment plants.

### Selection, Isolation, and Identification of Culturable MOB

Filter sands and PF gravels from LT and VO were also used as inoculum for bacterial isolation and selection of culturable MOB. A total of 202 colonies with different morphologies or brown colors, with potential Mn(II) oxidation activities were chosen from Lept, PC, and Mn culture media (Supplementary Table S4). The analysis of 16S rRNA gene sequences of these strains, revealed different profiles of bacterial genera isolated from VO and LT samples. Most of culturable MOB, from both water treatment plants were identified as *Pseudomonas*, a genus well-characterized as MOB (Geszvain et al., 2012). Also, an abundant proportion of *Flavobacterium* were found in LT; and *Sphingomonas* and *Acidovorax* in VO. Interestingly, several culturable MOB such as *Ensifer*, *Noviherbaspirillum*, *Sphingopyxis*, *Herpetosiphon*, *Legionella*, and *Chryseobacterium* have never been reported before as manganese oxidizers, and further studies will allow the investigation of new Mn(II) oxidation molecular mechanisms. Further, bacteria were identified using Matrix-assisted laser desorption ionization–time of flight mass spectrometry (MALDI-TOF MS) (Welker and Moore, 2011; Dingle and Butler-Wu, 2013). Using this approach, several *Flavobacterium* spp., *Legionella* spp., *Chryseobacterium* spp., and *Pseudomonas* spp. isolates were identified as potentially pathogenic bacterial species and, considering their potential role in the development of an inoculum, these isolates were excluded for further studies. Furthermore, *Streptomyces* spp., which reduces water quality because it produces odor; as well as *Sphingopyxis* spp., *Herpetosiphon* spp., *Acidovorax* spp*., Rhodobacter* spp. and *Cupriavidus* spp. that began to oxidize Mn(II) after 30 days of growth, were also discarded. Therefore, out of the total of 202 isolates, 23 were further characterized; these isolates were named as MOB followed by numbers denoting the position of the isolates in the strain collection (Table 1).

### Representation of the 23 Culturable MOB in the Total Bacteria Identified by Illumina Sequencing

**FIGURE 2:**
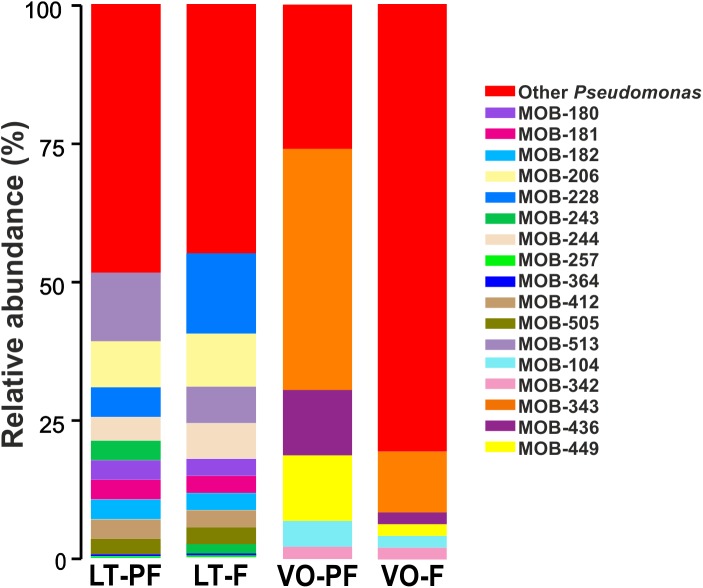
Proportion of cultivated *Pseudomonas* in LT and VO pre-filters (PFs) and filters. Bar-charts show abundance of cultivated *Pseudomonas* relative to total *Pseudomonas* OTUs found by Illumina sequencing. Red bars indicate the percentage of other *Pseudomonas* not cultivated in this work.

In order to determine the proportion in which the 23 cultured MOB (Table 1) were present in the total bacterial community, Sanger sequences of the 23 MOB 16S rRNA gene were compared with all 16S rRNA gene sequences obtained from LT and VO samples by Illumina sequencing. The cultured strains *Noviherbaspirillum* sp., *Terrabacter* sp., *Ensifer* sp., and *Rhizobium* sp. were not found by Illumina sequencing, suggesting that even if these bacteria are present in low quantities, they can be easily cultured under laboratory conditions. Cultured *Sphingomonas* sp. were found to represent 0.2% of the bacterial community in LT PF and 0.9% in VO filter (Supplementary Table S2). *Pseudomonas* OTUs abundance, obtained by Illumina sequencing, was 5.9% in LT PF and 1.6% in LT filter (Supplementary Table S2). From these OTUs, 51.1% from LT PF and 56.2% from LT filter corresponded to OTUs that matched with 16S rRNA gene sequences of the *Pseudomonas* that were culturable in this work (Figure 2). In VO, *Pseudomonas* represented 0.3% in both VO PF and VO filter according to Illumina sequencing data (Supplementary Table S2). From these reads the culturable MOB represented nearly 74% in VO PF and 20% in VO filter (Figure 2).

Overall cultured MOB represented a low proportion of total bacteria present in both water treatment plants. These results suggest two possibilities: the drivers of Mn(II) oxidation in the biofilters could be minor members of the total bacterial community or there are non-culturable bacteria that are the major members playing a role in Mn(II) oxidation process.

### Selection of MOB With the Highest Mn(II) Oxidation Capacities

In order to select the best MOB applicable to formulate a bacterial inoculum to improve Mn(II) removal, quantifications of their Mn(II) oxidation capacities were performed. Quantifications were performed in the culture media where the bacteria were selected (Mn, PC, or Lept Media). Results showed that Mn(II) oxidation capacity was time-dependent and that it was different among the isolated strains (Supplementary Figure S4). Concerning the strains selected in PC-medium, *Noviherbaspirillum* MOB-109, and *Pseudomonas* MOB-436 and MOB-343 demonstrated the highest Mn(II) oxidation capacities, reaching the maximum at 7, 11, and 15 days, respectively. In Lept-medium, *Pseudomonas* MOB-180, MOB-181 and MOB-182 showed the highest Mn(II) oxidizing capacity at shorter times of growth (4 days). Due to the marked similarity among them, only one of these strains, MOB-181, was fully characterized in this study. On the other hand, *Pseudomonas* MOB-449 revealed the next highest oxidation capacity at 20 days. Among other bacterial genera, *Terrabacter* MOB-326 showed a good performance of Mn(II) oxidation. MOB assayed in Mn-medium showed the lowest Mn(II) oxidation capacity. Therefore, on the basis of their high Mn(II) oxidizing capacities and in order to select different bacterial species, MOB-109, MOB-181, MOB-326, MOB-343, MOB-436, and MOB-449 were chosen for further characterization (Supplementary Figure S4).

### Assessment of Biofilm Formation by Selected MOB Strains

**FIGURE 3:**
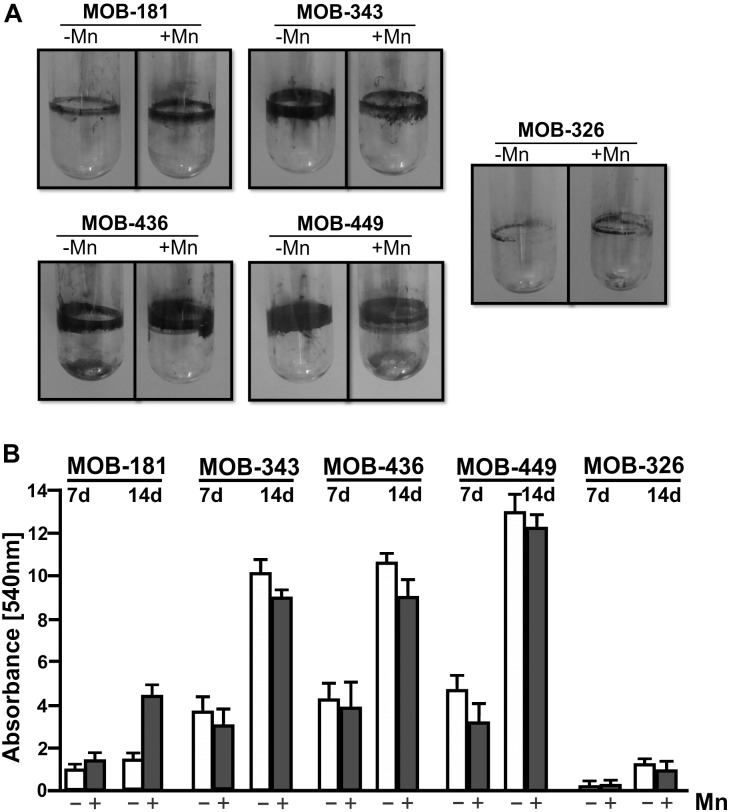
Biofilm assays for the selected MOB. **(A)** Representative photographs of CV stained bacterial biofilms. Five bacterial isolates were grown statically in LB medium in absence or presence of 100 μM of MnSO_4_ (–Mn or +Mn, respectively) at 28°C in borosilicate glass tubes for 14 days. **(B)** Biofilms quantifications were performed by CV staining (7 and 14 days), measured spectrophotometrically (Abs. at 540 nm). Values represent the mean from measurements done in triplicate. Error bars indicate the SD. Significance: *P* < 0.05.

Biofilm formation was evaluated for the six chosen strains. *Pseudomonas* strains had the ability to form large and dense biofilms, developing rings at the air–liquid surface–interface of the culture medium, while biofilms formed by *Terrabacter* MOB-326 were significantly lower in size and density (Figure 3A). *Noviherbaspirillum* MOB-109 strain was unable to form biofilms in both conditions (data not shown). *Pseudomonas* MOB-449 strain showed the greatest ability to form biofilms (Figure 3A). These results were corroborated by biofilm quantifications at 7 and 14 days through measurements of CV staining (Figure 3B). Since the isolates should grow in the presence of Mn, bacterial biofilm formation was also assayed in the presence of this metal. Biofilm formation capacity of MOB-181 was increased by addition of Mn (*P* < 0.05) (Figure 3B). MOB-449 also could form biofilm at 18°C (Supplementary Figure S5) that is its optimal oxidation temperature (see below). This assay revealed that MOB-181, MOB-326, MOB-343, MOB-436, and MOB-449 can attach to solid surfaces, thus confirming their potential for biotechnological applications in water sand filters. MOB-109 was excluded from further analysis because it was unable to form biofilms under conditions that regularly support biofilm formation.

### Influence of Temperature on the Mn(II) Oxidizing Capacity of Selected MOB Strains

**FIGURE 4:**
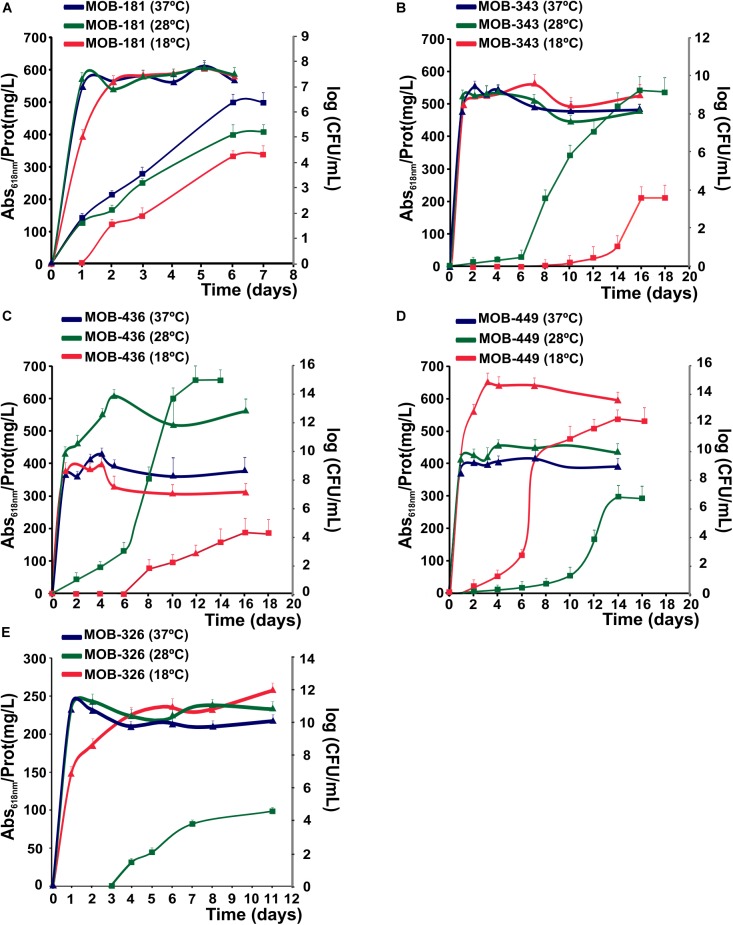
Effect of temperature on bacterial growth and Mn(II) oxidation capacities of the selected MOB at 18, 28, and 37°C, represented in red, green and blue, respectively. **(A)** MOB-181, **(B)** MOB-343, **(C)** MOB-436, and **(D)** MOB-449 in PC-medium and **(E)** MOB-326 grown in Lept-medium. Mn(II) oxidation capacities are represented by squares. The axes on the right side of the plot (gray color) represent the bacterial population at the different temperatures (as log CFU/mL) as a function of time (triangles). Values represent the mean from measurements done in triplicate for Mn(II) oxidation and bacterial population at each time point analyzed. Error bars indicate the SD. Significance: *P* < 0.05.

The effect of temperature on the kinetics of biological Mn(II) oxidation is especially relevant and important for filtration systems because they are exposed to seasonal and diurnal temperature variations. Therefore, Mn(II) oxidation and bacterial growth at 18, 28, and 37°C were quantified at different times. All strains could grow at three assayed temperatures; and as described previously for different MOB (Tebo et al., 2005), Mn(II) oxidation activity began at stationary phase (Figure 4). MOB-181 presented the maximal Mn(II) oxidation capacity at 37°C (*P* < 0.05); MOB-343, MOB-436, and MOB-326 at 28°C (*P* < 0.05); and MOB-449 at 18°C (*P* < 0.05) (Figure 4). MOB-181, MOB-343, and MOB-326 grown similar at all temperatures, however, only MOB-181 was capable of oxidizing Mn(II) at all temperatures (Figure 4A,C,D). These results suggest that different molecular mechanism or differential gene expression trigger Mn(II) oxidation in these bacteria. A correlation between bacterial growth and Mn(II) oxidation performances was observed for MOB-436 and MOB-449. For these bacteria, optimal growth temperatures (28°C for MOB-436 and 18°C for MOB-449) were the same to optimal Mn(II) oxidation temperatures. The different results found between strains related to Mn(II) oxidation and growth at different temperatures make these bacteria excellent models to study the physiological role of Mn(II) oxidation and the molecular mechanism involved in this process.

These results indicate that MOB-181 was the most versatile strain since it could oxidize Mn(II) at all temperatures and MOB-449 showed the highest Mn(II) oxidation capacity at lower temperatures, suggesting that this strain could be relevant for the optimization of biological Mn(II) removal processes at cold months. Therefore, both MOB-181 and MOB-449 may be appropriate candidates to design a bacterial inoculum for Mn(II) removal at different seasons.

### Mn(II) Oxidation Variability in the Presence of Different Concentrations of Mn(II) and Fe(II) in the Culture Medium

**FIGURE 5:**
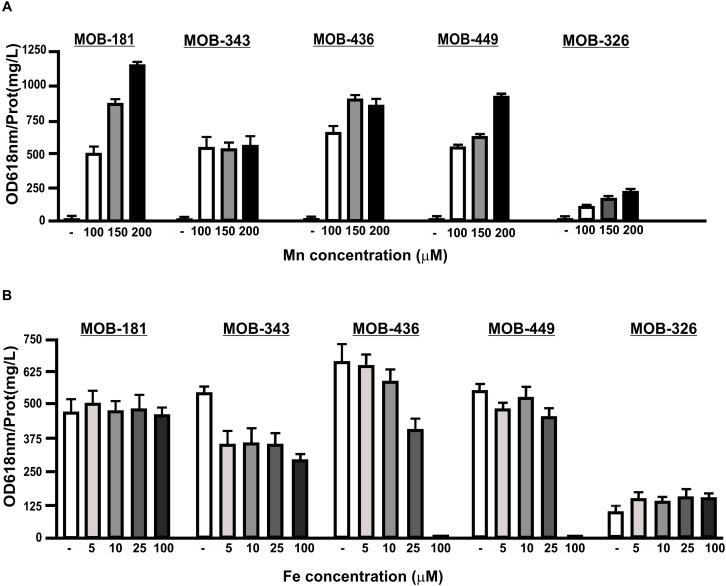
The influence of different Mn(II) or Fe(II) concentrations on Mn(II) oxidation capacities of the selected MOB. Mn(II) oxidation was quantified for the strains incubated with increasing concentrations of **(A)** MnSO_4_ (100, 150, and 200 μM) or **(B)** 100 μM MnSO_4_ and FeSO_4_ (5, 10, 25, and 100 μM) in PC-medium for MOB-181, MOB-343, MOB-436, and MOB-449 and in Lept-medium for MOB-326. Quantifications were performed at the optimal Mn(II) oxidation temperature and at the time where Mn(II) oxidation was the highest for each strain (see Figure 4). Values represent the mean from measurements done by triplicate. Error bars indicate the SD. Significance: *P* < 0.05.

Manganese-oxidizing bacteria Mn(II) oxidation capacities were evaluated in the presence of different concentrations of MnSO_4_. All strains were able to oxidize Mn(II) at higher concentrations of this metal (up to 200 μM). For MOB-181, MOB-326, and MOB-449, the higher Mn(II) concentrations in the medium, the better oxidation capacities were obtained (*P* < 0.05) (Figure 5A). Although Mn(II) oxidation was not inhibited in the presence of higher Mn concentrations, MOB-343 reached the maximum for this activity at 100 μM MnSO_4_ concentration and MOB-436 at 150 μM MnSO_4_ (Figure 5A). These results reinforce that MOB-181 and MOB-449 are the best candidates for bacterial inoculum development. Presence of Fe(II) concomitantly with Mn(II) is frequently observed in groundwater (Pacini et al., 2005). Therefore, analyses to determine if Fe(II) can inhibit Mn(II) oxidation were performed. Supplementation with Fe(II), did not affect Mn(II) oxidation performed of MOB-181 and MOB-326 strains (Figure 5B). Mn(II) oxidation was inhibited by Fe(II) 100 μM in MOB-436 and MOB-449, and this activity was reduced at all Fe(II) concentration assayed for MOB-343 strain (Figure 5B). These results showed that all strains were able to oxidize Mn(II) in the presence of Fe(II) at the concentrations frequently found in groundwater (∼25 μM, Supplementary Table S1).

### MOB Growth Interaction Assays

The presence of a bacterium that could impair another bacterial fitness would be inappropriate for the ultimate goal of using these strains to perform mixed inoculums. Therefore, *in vitro* growth interactions of the five selected strains were carried out. In the presence of MOB-343 strain, great inhibitory effects on growth and survival of MOB-326, MOB-436, and MOB-449 strains were observed, evidenced by inhibition halos developed (Supplementary Figure S6). These results indicated that MOB-343 should be avoided in mixed inoculums since it inhibits the growth of other bacteria.

### Groundwater Mn(II) Oxidation by Immobilized MOB

**FIGURE 6:**
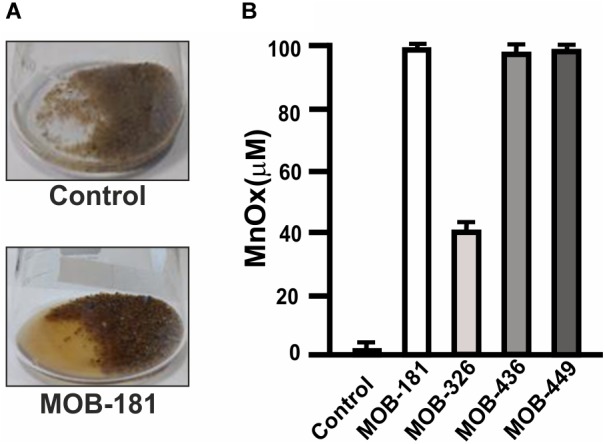
Groundwater Mn(II) oxidation performed by bacterial inoculated sands. **(A)** Representative photograph of Mn(II) oxidation in groundwater containing 100 μM MnCl_2_ by sand inoculated with MOB-181 strain (bottom panel) and of the lack of Mn(II) oxidation in sands not inoculated with bacteria used as control (upper panel). **(B)** The concentration of Mn oxides (MnOx) formed by sand immobilized MOB after 7 days of incubation was determined. Three independent experiments were performed and the mean values (bars) and the SD (error bars) are shown. Significance: *P* < 0.05.

Groundwater Mn(II) removal processes require bacteria that can oxidize Mn(II) attached to filtering material. Therefore, MOB were immobilized on sands and the capacity to oxidize 100 μM Mn(II) present in groundwater was analyzed. All strains produced brownish colorations in groundwater due to Mn oxides formation. As an example, Mn(II) oxidation performed by MOB-181 strain is shown (Figure 6A). Concentration of Mn oxides (MnOx) formed in groundwater revealed that strains oxidized all the Mn(II) that was present in groundwater (100 μM), except for MOB-326 which achieved a maximal MnOx concentration of 40 μM MnOx (Figure 6B). This may be due to the lower capacity of MOB-326 to grow in biofilms (Figure 4). There was no evidence of groundwater Mn oxidation in the cell free control (Figure 6A,B). These results indicate that MOB-181, MOB-436, and MOB-449 may be immobilized on filtering material and that these strains are highly efficient to oxidize Mn(II) present in groundwater.

## Conclusion

Biological filtration is used to remove metals present in groundwater since it is a cost-effective technology that minimizes the use of chemical oxidants. However, in these biological processes an optimal Mn(II) removal may only be achieved in systems having appropriate microbial communities (Mouchet, 1992; Vandenabeele et al., 1992; Katsoyiannis and Zouboulis, 2004; Burger et al., 2008a; Tekerlekopoulou et al., 2008). In this work, in order to characterize novel bacteria with these properties, bacterial communities and culturable MOB residing in two groundwater biological treatment plants were studied. A summary of the Mn(II) oxidation performance evaluated for the characterized MOB is shown in Table 2. There is strong evidence to postulate MOB-181 and MOB-449 as the best potential candidates to make a bacterial inoculum applicable to improve Mn(II) removal processes. These strains were abundant in the filtration systems since they were almost 4 and 12% of the total *Pseudomonas* spp. detected by Illumina sequencing in samples from which they were cultured. Both strains were able to oxidize Mn(II) in different assayed growth media and up to 200 μM. They were also able to oxidize Mn(II) at different temperatures, were strong biofilm formers and could oxidize Mn(II) while immobilized on sand (Table 2). Overall, this work contributes to the knowledge of bacterial communities present in biological water treatment plants and will help in designing appropriate bacterial inoculums that, in the near future, would allow the optimization of Mn removal from groundwater.

**Table 2:** Summary of the effect of growth in different culture media, temperature and metals on Mn(II)-oxidation capacities of selected MOB.

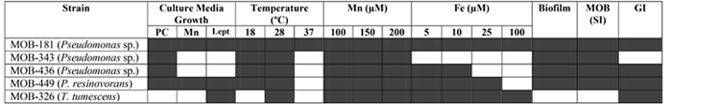

****Culture media and temperature:****gray indicates that the MOB can oxidize Mn(II) in the indicated media (PC, Mn, or Lept) or temperature (18, 28, or 37°C) and white that MOB lost Mn(II)-oxidation capacity in this media or temperature.****Mn:****gray indicates that the MOB can oxidize Mn(II).****Fe:****gray indicates that the MOB can oxidize Mn(II) and white that Mn(II)-oxidation was reduced at this Fe(II) concentration.****Biofilm:****Gray indicates that the bacteria can form thick biofilms and white that the bacteria form narrow biofilms.****MOB immobilized on sand (SI):****Gray indicates that the MOB immobilized on sand can oxidize all the Mn(II) present in groundwater, white indicates a partial Mn(II)-oxidation.****Growth interaction (GI):****Gray indicates that the MOB do not inhibit the growth of the other isolates, white indicates the contrary*.*

## Author Contributions

VP, GS, JO, and NG designed the experiments and wrote the manuscript. AP and LCC performed the experimental work. All authors reviewed and corrected the manuscript.

## Conflict of Interest Statement

The authors declare that the research was conducted in the absence of any commercial or financial relationships that could be construed as a potential conflict of interest.

## Footnotes

1 http://www.bioinformatics.babraham.ac.uk/projects/fastqc/

2 http://multiqc.info/

3 http://sco.h-its.org/exelixis/web/software/pear/

4 http://hannonlab.cshl.edu/fastx_toolkit/

5 https://sourceforge.net/projects/bbmap/

6 https://github.com/torognes/vsearch

7 https://rdp.cme.msu.edu/

8 http://qiime.org/

9 http://bioinfo.lifl.fr/RNA/sortmerna/

10 https://git.metabarcoding.org/obitools/sumatra/wikis/home

11 https://www.indear.com/

## Abbreviations

F: filter
LT: Las Toscas
MOB: manganese-oxidizing bacteria
PF: pre-filter
VO: Villa Ocampo
