# Supplementary material for: Environmental Bacteria Involved in Manganese(II) Oxidation and Removal From Groundwater
Source: Front Microbiol. 2019 Feb 11;10:119. doi: 10.3389/fmicb.2019.00119 (PMC6396730; doi:10.3389/fmicb.2019.00119)
Supplement: Supplementary file 1 [file Data_Sheet_1.PDF]

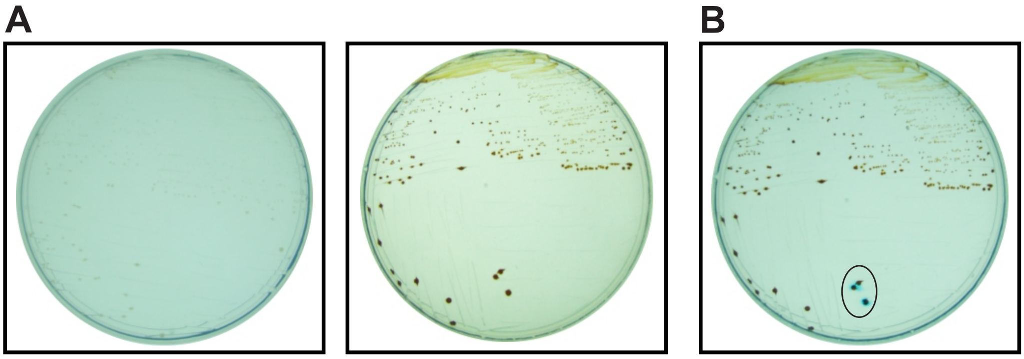

Supplementary Figure 1. Selection of the MOB on different media.
A. Representative plates showing how MOB were identified by the detection of dark color of the colonies due to Mn-oxides precipitated around or near the cells in media supplemented with 100 μM $MnSO_4$ (right plate). In the absence of $MnSO_4$the colonies on the agar remained whitish (left plate).
B. To confirm that the brown color corresponded to Mn-oxides, LBB staining of the colonies was performed; the blue color indicated the presence of Mn-oxides.

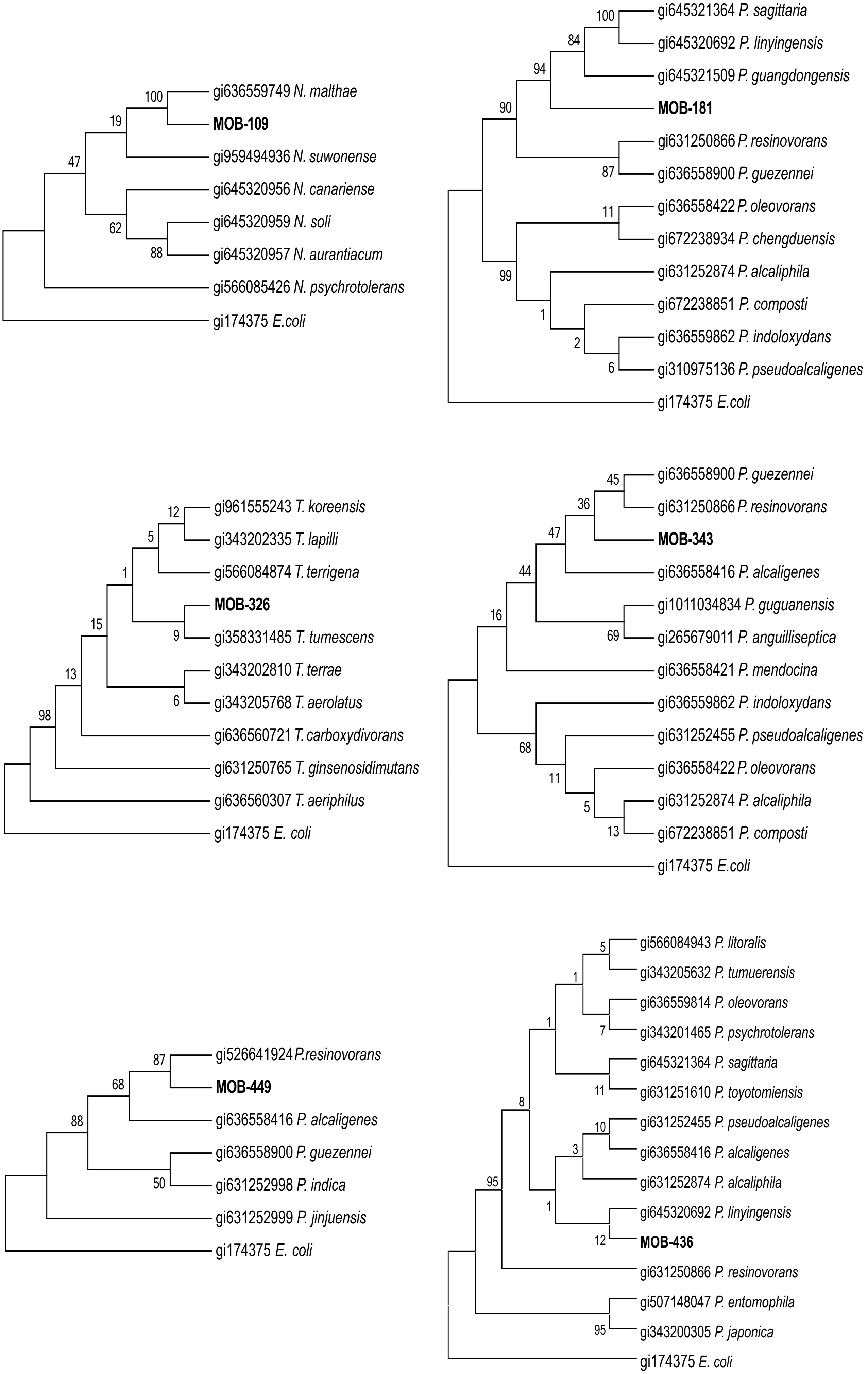

Supplementary Figure 2. Phylogenetic trees based on the 16S rRNA sequences. Phylogenetic trees were constructed with the 16S rRNA sequences. The trees shown correspond to the six isolates that were further characterized in this work.

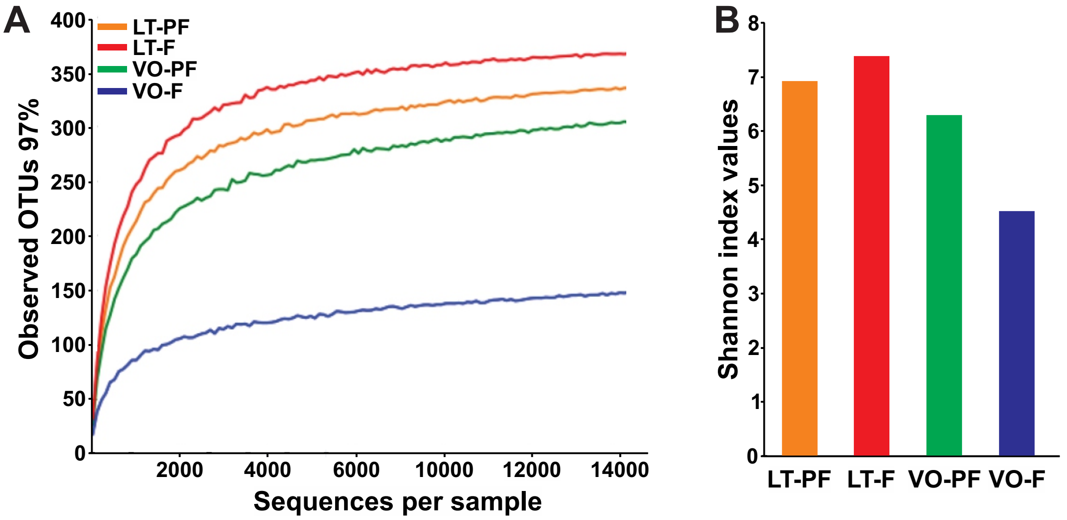

Supplementary Figure 3. Alpha diversity metrics derived from 16S rRNA gene sequences of bacteria associated with LT and VO samples. Sequences with similarity > 97% were treated as Operational Taxonomic Units (OTU-97%) and considered as "closely related" species. **(A)** Rarefaction curves. Bacterial alpha diversity, measured as OTU richness, was estimated for PF and F from LT and VO microbiomes (referred as LT-PF, LT-F, VO-PF and VO-F). Rarefaction curves of all microbiomes tended to an asymptote indicating that the sequencing depth was enough for an exhaustive exploration of the bacterial communities present in these fractions. **(B)** Shannon diversity of bacteria from each LT and VO samples. The bacterial diversity, estimated by the Shannon diversity index from the OTU data, was highest for LT-F samples followed by LT-P, VO-PF and the lowest value was observed in VO-F.

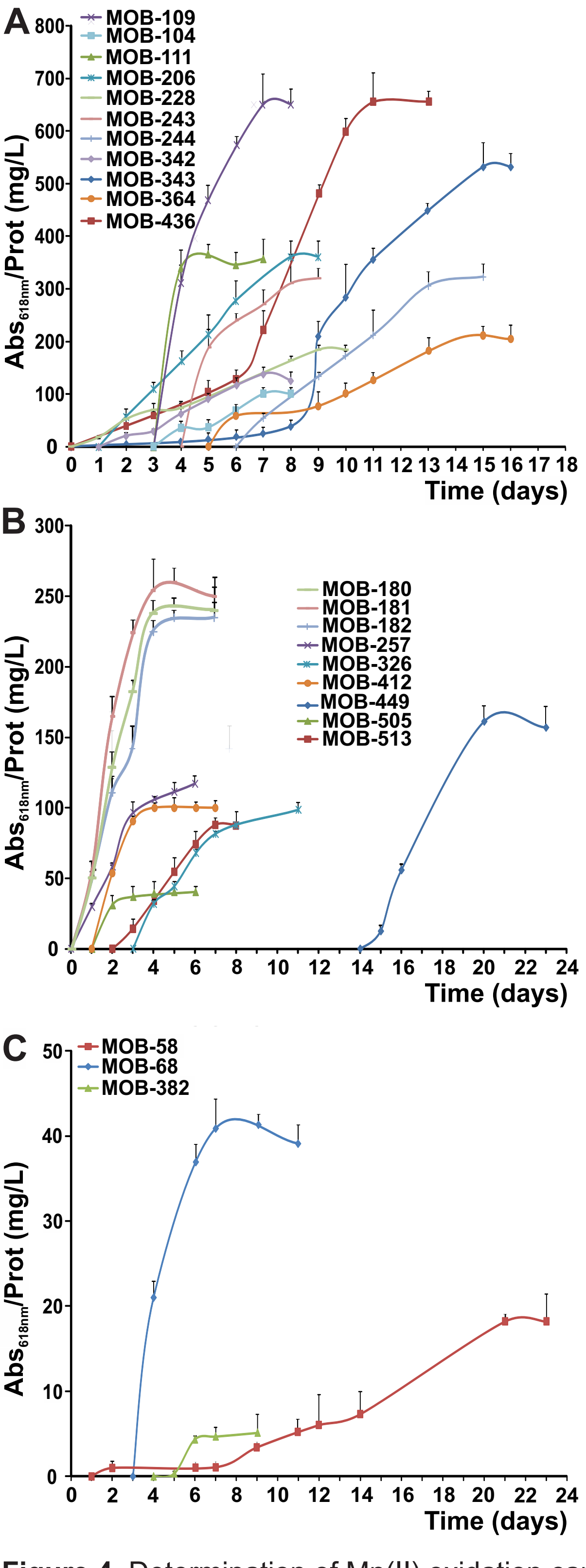

Supplementary Figure 4. Determination of Mn(II) oxidation capacities of the different bacterial spots grown on solid media.

A. Quantification of Mn(II) oxidation by the LBB assay at different times for the bacteria isolated in PC-medium. Three independent experiments were performed and the mean values and the standard deviations (SD, error bars) are shown.

B. Quantification of Mn(II) oxidation for the bacteria isolated in Lept-medium. Experiments and statistical analysis were performed as indicated above (A).

C. Quantification of Mn(II) oxidation for the bacteria isolated in Mn-Medium. Experiments and statistical analysis were performed as indicated above for (A).

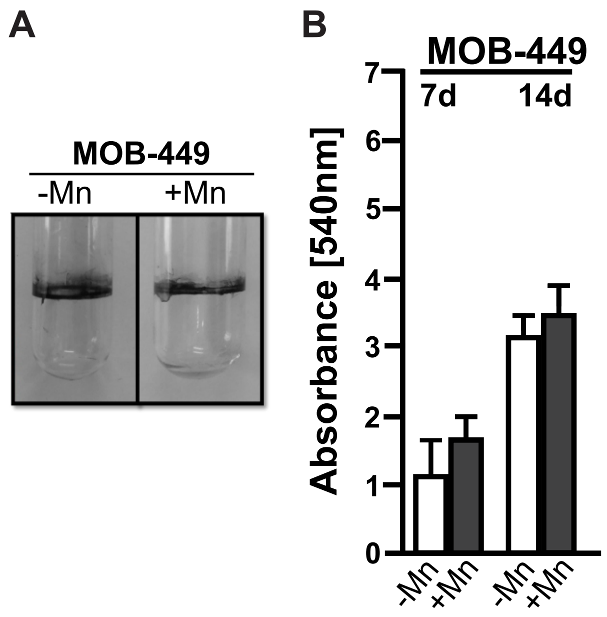

Supplementary Figure 5. Biofilm assays for MOB-449 at 18ºC.

A. Representative photographs of CV stained bacterial biofilm of MOB-449 grown statically LB medium in presence or absence of 100 μM of $MnSO_4$ (-Mn or +Mn, respectively) at 18ºC in borosilicate glass tubes for 14 days.

B. Biofilms quantifications were performed by CV staining (7 and 14 days), measured spectrophotometrically (Abs. at 540 nm). Values represent the mean from measurements done in triplicate. Error bars indicate the SD. Significance: $p< 0.05$.

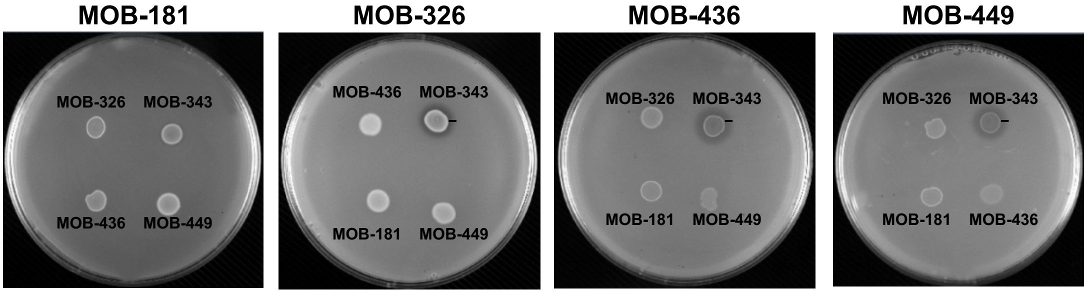

Supplementary Figure 6. Growth interactions assay.
Inhibition of the growth of one strain by another was detected by the appearance of inhibition halos. Upper to the panels the names of the strains that were spread onto LB agar are indicated. Black line indicates the inhibitory growth zone around MOB-343 strain spots.

Supplementary Table 1. LT and VO groundwater and effluent water physico-chemical characteristics.

Compound	LT-Groundwater	LT-Effluent water	VO-Groundwater	VO-Effluent water
Temperature (ºC)	26 ± 0.4	26 ± 0.3	26 ± 0.3	26 ± 0.3
pH	6.6 ± 0.2	6.5 ± 0.2	6.4 ± 0.1	6.6 ± 0.1
Manganese (mg/L)	0.43* ± 0.05	0.049 ± 0.001	0.33* ± 0.07	0.053 ± 0.001
Iron (mg/L)	1.27* ± 0.08	0.11 ± 0.03	1.61* ± 0.06	0.13 ± 0.04
Nitrite (mg/L)	0.006 ± 0.001	0.006 ± 0.001	0.006 ± 0.001	0.006 ± 0.001
Nitrate (mg/L)	1.2 ± 0.1	1.1 ± 0.1	0.9 ± 0.2	0.7 ± 0.2
Sulfate (mg/L)	304 ± 0.8	302 ± 0.9	49 ± 0.9	51 ± 0.9
Bicarbonate (mg/L)	379 ± 4	375 ± 4	312 ± 3	318 ± 5
Fluoride (mg/L)	1.1 ± 0.05	0.9 ± 0.05	0.2 ± 0.002	0.14 ± 0.01
Sodium (mg/L)	644 ± 1	644 ± 1	207 ± 2	161 ± 2
Chloride (mg/L)	476 ± 2	202 ± 7	405 ± 5	200 ± 7
Calcium (mg/L)	85 ± 6	81 ± 4	131 ± 4	136 ± 5
Magnesium (mg/L)	46 ± 1	42 ± 1	17 ± 0.8	19 ± 0.7

* Mn concentration was equal to 8 μM and 6 μM LT-Groundwater and VO-Groundwater, respectively. Fe concentration was equal to 23 μM and 28 μM LT-Groundwater and VO-Groundwater, respectively.

Supplementary Table 2. Abundance and distribution of all bacterial classes found in LT-PF, LT-F, VO-PF and VO-F samples. All sequences were taxonomically assigned by BLASTN against NCBI 16S rRNA genes and were classified to classes (>90% similarity to best-hit). Sequences were grouped by sample, site and classification and relative abundances were calculated according to total number of sequences per sample.

	Total	LT-PF	LT-F	VO-PF	VO-F
Taxonomy	%	%	%	%	%
Unassigned;Other;Other	2.10%	2.30%	3.80%	1.10%	1.20%
k__Bacteria;p__Acidobacteria;c__Acidobacteria-6	0.20%	0.00%	0.00%	0.60%	0.10%
k__Bacteria;p__Acidobacteria;c__Holophagae	1.00%	0.10%	0.10%	3.80%	0.10%
k__Bacteria;p__Acidobacteria;c__RB25	0.10%	0.00%	0.00%	0.30%	0.00%
k__Bacteria;p__Acidobacteria;c__Solibacteres	0.20%	0.10%	0.20%	0.10%	0.60%
k__Bacteria;p__Acidobacteria;c__Sva0725	0.10%	0.00%	0.00%	0.30%	0.00%
k__Bacteria;p__Acidobacteria;c__[Chloracidobacteria]	2.40%	2.60%	1.30%	1.60%	4.00%
k__Bacteria;p__Armatimonadetes;c__[Fimbriimonadia]	0.20%	0.50%	0.20%	0.00%	0.00%
k__Bacteria;p__Bacteroidetes;c__Bacteroidia	0.40%	0.00%	1.70%	0.00%	0.00%
k__Bacteria;p__Bacteroidetes;c__Cytophagia	0.80%	1.60%	0.90%	0.60%	0.00%
k__Bacteria;p__Bacteroidetes;c__Flavobacteriia	0.10%	0.30%	0.30%	0.00%	0.00%
k__Bacteria;p__Bacteroidetes;c__Sphingobacteriia	1.10%	0.90%	0.70%	1.00%	2.00%
k__Bacteria;p__Bacteroidetes;c__[Saprospirae]	0.90%	1.40%	1.10%	1.20%	0.10%
k__Bacteria;p__Chlamydiae;c__Chlamydiia	1.00%	0.00%	0.00%	1.10%	2.80%
k__Bacteria;p__Chloroflexi;c__Anaerolineae	0.20%	0.10%	0.10%	0.80%	0.00%
k__Bacteria;p__Chloroflexi;c__Chloroflexi	1.00%	1.40%	0.20%	2.30%	0.00%
k__Bacteria;p__Chloroflexi;c__TK17	0.00%	0.00%	0.00%	0.10%	0.00%
k__Bacteria;p__Cyanobacteria;c__4C0d-2	0.10%	0.10%	0.10%	0.00%	0.20%
k__Bacteria;p__Cyanobacteria;c__Chloroplast	1.20%	0.80%	2.20%	2.00%	0.00%
k__Bacteria;p__Cyanobacteria;c__ML635J-21	0.30%	0.60%	0.50%	0.00%	0.00%
k__Bacteria;p__Cyanobacteria;c__Oscillatoriophycideae	1.10%	0.10%	1.20%	3.10%	0.00%
k__Bacteria;p__Cyanobacteria;c__Synechococcophycideae	0.10%	0.00%	0.00%	0.20%	0.00%

k__Bacteria;p__Elusimicrobia;c__Elusimicrobia	0.10%	0.20%	0.10%	0.00%	0.00%
k__Bacteria;p__Firmicutes;c__Clostridia	0.60%	0.10%	2.40%	0.00%	0.00%
k__Bacteria;p__Firmicutes;c__Erysipelotrichi	0.20%	0.00%	0.70%	0.00%	0.00%
k__Bacteria;p__Fusobacteria;c__Fusobacteriia	0.40%	0.10%	1.50%	0.00%	0.00%
k__Bacteria;p__GN02;c__	0.00%	0.10%	0.10%	0.00%	0.00%
k__Bacteria;p__GN02;c__BD1-5	0.20%	0.20%	0.50%	0.00%	0.00%
k__Bacteria;p__GN04;c__GN15	0.10%	0.00%	0.10%	0.20%	0.00%
k__Bacteria;p__GN04;c__MSB-5A5	0.00%	0.00%	0.00%	0.20%	0.00%
k__Bacteria;p__Gemmatimonadetes;c__Gemm-1	0.10%	0.10%	0.20%	0.00%	0.00%
k__Bacteria;p__NKB19;c__	0.10%	0.10%	0.10%	0.20%	0.20%
k__Bacteria;p__Nitrospirae;c__Nitrospira	15.9%	7.60%	5.80%	24.9%	25.4%
k__Bacteria;p__OD1;c__	0.10%	0.00%	0.00%	0.20%	0.00%
k__Bacteria;p__OP3;c__PBS-25	0.00%	0.10%	0.10%	0.00%	0.00%
k__Bacteria;p__Planctomycetes;c__OM190	0.20%	0.20%	0.10%	0.70%	0.00%
k__Bacteria;p__Planctomycetes;c__Phycisphaerae	0.90%	1.30%	1.10%	1.00%	0.30%
k__Bacteria;p__Planctomycetes;c__Planctomycetia	2.00%	1.10%	1.10%	3.30%	2.60%
k__Bacteria;p__Planctomycetes;c__[Brocadiae]	0.00%	0.00%	0.00%	0.10%	0.00%
k__Bacteria;p__Planctomycetes;c__vadinHA49	0.20%	0.00%	0.20%	0.30%	0.10%
k__Bacteria;p__Proteobacteria;c__Alphaproteobacteria	9.00%	11.6%	7.80%	7.00%	9.70%
k__Bacteria;p__Proteobacteria;c__Betaproteobacteria	28.50%	34.10%	43.40%	25.20%	11.10%
k__Bacteria;p__Proteobacteria;c__Deltaproteobacteria	6.00%	2.20%	7.00%	4.60%	9.90%
k__Bacteria;p__Proteobacteria;c__Epsilonproteobacteria	0.50%	1.50%	0.40%	0.10%	0.00%
k__Bacteria;p__Proteobacteria;c__Gammaproteobacteria	12.40%	25.10%	9.40%	9.60%	5.50%
k__Bacteria;p__Proteobacteria;c__Zetaproteobacteria	0.10%	0.00%	0.00%	0.50%	0.00%
k__Bacteria;p__SBR1093;c__VHS-B5-50	0.10%	0.00%	0.00%	0.10%	0.00%
k__Bacteria;p__TM7;c__TM7-1	0.10%	0.00%	0.00%	0.20%	0.00%
k__Bacteria;p__Verrucomicrobia;c__Opitutae	6.90%	0.80%	2.40%	0.50%	24.0%
k__Bacteria;p__Verrucomicrobia;c__Verrucomicrobiae	0.00%	0.10%	0.10%	0.00%	0.00%

k__Bacteria;p__Verrucomicrobia;c__[Pedosphaerae]	0.50%	0.50%	1.00%	0.40%	0.20%
k__Bacteria;p__WS3;c__PRR-12	0.10%	0.00%	0.10%	0.10%	0.00%

Supplementary Table 3. Abundance and distribution of all bacterial genera found in LT-PF, LT-F, VO-PF and VO-F samples. All sequences were taxonomically assigned by BLASTN against NCBI 16S rRNA and were classified to genus (>90% similarity to best-hit), family or order (depending on the case for hits <90% similarity to best-hit). Sequences were grouped by sample, site and classification and relative abundances were calculated according to total number of sequences per sample. The genera that were cultivated and characterized as MOB are highlighted in pink color.

	**Total**	**LT-PF**	**LT-F**	**VO-PF**	**VO-F**
**Legend Taxonomy**	%	%	%	%	%
Unassigned;Other;Other;Other;Other;Other	2.3%	2.1%	3.8%	1.1%	1.2%
k__Bacteria;p__Acidobacteria;c__Acidobacteria-6;o__iii1-15;f__;g__	0.1%	0.0%	0.0%	0.5%	0.0%
k__Bacteria;p__Acidobacteria;c__Acidobacteria-6;o__iii1-15;f__mb2424;g__	0.1%	0.0%	0.0%	0.2%	0.1%
k__Bacteria;p__Acidobacteria;c__Holophagae;o__Holophagales;f__;g__	1.0%	0.1%	0.1%	3.8%	0.1%
k__Bacteria;p__Acidobacteria;c__RB25;o__;f__;g__	0.1%	0.0%	0.0%	0.3%	0.0%
k__Bacteria;p__Acidobacteria;c__Solibacteres;o__Solibacterales;f__;g__	0.2%	0.1%	0.2%	0.1%	0.3%
k__Bacteria;p__Acidobacteria;c__Solibacteres;o__Solibacterales;f__[Bryobacteraceae]	0.1%	0.0%	0.0%	0.0%	0.4%
k__Bacteria;p__Acidobacteria;c__Sva0725;o__Sva0725;f__;g__	0.1%	0.0%	0.0%	0.3%	0.0%
k__Bacteria;p__Acidobacteria;c__[Chloracidobacteria];o__PK29;f__;g__	0.1%	0.0%	0.0%	0.3%	0.0%
k__Bacteria;p__Acidobacteria;c__[Chloracidobacteria];o__RB41;f__Ellin6075;g__	2.3%	2.6%	1.3%	1.4%	4.0%
k__Bacteria;p__Armatimonadetes;c__[Fimbriimonadia];o__[Fimbriimonadales];f__;g__	0.2%	0.5%	0.2%	0.0%	0.0%
k__Bacteria;p__Bacteroidetes;c__Bacteroidia;o__Bacteroidales;f__Marinilabiaceae;g__	0.2%	0.0%	0.7%	0.0%	0.0%
k__Bacteria;p__Bacteroidetes;c__Bacteroidia;o__Bacteroidales;f__Porphyromonadaceae;g__Paludibacter	0.1%	0.0%	0.4%	0.0%	0.0%
k__Bacteria;p__Bacteroidetes;c__Bacteroidia;o__Bacteroidales;f__Rikenellaceae;g__Blvii28	0.2%	0.0%	0.6%	0.0%	0.0%
k__Bacteria;p__Bacteroidetes;c__Cytophagia;o__Cytophagales;f__Cyclobacteriaceae;g__	0.3%	1.0%	0.2%	0.0%	0.0%
k__Bacteria;p__Bacteroidetes;c__Cytophagia;o__Cytophagales;f__Cytophagaceae;g__	0.3%	0.2%	0.6%	0.6%	0.0%
k__Bacteria;p__Bacteroidetes;c__Cytophagia;o__Cytophagales;f__Cytophagaceae;g__Flectobacillus	0.1%	0.4%	0.1%	0.0%	0.0%
k__Bacteria;p__Bacteroidetes;c__Flavobacteriia;o__Flavobacteriales;f__Flavobacteriaceae;g__	0.1%	0.1%	0.2%	0.0%	0.0%
k__Bacteria;p__Bacteroidetes;c__Flavobacteriia;o__Flavobacteriales;f__Flavobacteriaceae;g__Flavobacterium	0.1%	0.2%	0.1%	0.0%	0.0%
k__Bacteria;p__Bacteroidetes;c__Sphingobacteriia;o__Sphingobacteriales;f__;g__	1.1%	0.9%	0.7%	1.0%	2.0%
k__Bacteria;p__Bacteroidetes;c__[Saprospirae];o__[Saprospirales];f__Chitinophagaceae;g__	0.2%	0.4%	0.5%	0.0%	0.0%
k__Bacteria;p__Bacteroidetes;c__[Saprospirae];o__[Saprospirales];f__Chitinophagaceae;g__Sediminibacterium	0.3%	0.6%	0.2%	0.5%	0.1%
k__Bacteria;p__Bacteroidetes;c__[Saprospirae];o__[Saprospirales];f__Saprospiraceae;g__	0.2%	0.2%	0.1%	0.6%	0.0%
k__Bacteria;p__Bacteroidetes;c__[Saprospirae];o__[Saprospirales];f__Saprospiraceae;g__Haliscomenobacter	0.1%	0.2%	0.3%	0.1%	0.0%
k__Bacteria;p__Chlamydiae;c__Chlamydiia;o__Chlamydiales;f__;g__	0.2%	0.0%	0.0%	1.0%	0.0%
k__Bacteria;p__Chlamydiae;c__Chlamydiia;o__Chlamydiales;f__Parachlamydiaceae;g__Parachlamydia	0.0%	0.0%	0.0%	0.1%	0.0%
k__Bacteria;p__Chlamydiae;c__Chlamydiia;o__Chlamydiales;f__Rhabdochlamydiaceae;g__Candidatus Rhabdochlamydia	0.7%	0.0%	0.0%	0.0%	2.7%
k__Bacteria;p__Chloroflexi;c__Anaerolineae;o__H39;f__;g__	0.2%	0.1%	0.1%	0.8%	0.0%
k__Bacteria;p__Chloroflexi;c__Chloroflexi;o__AKIW781;f__;g__	0.1%	0.3%	0.0%	0.0%	0.0%

k__Bacteria;p__Chloroflexi;c__Chloroflexi;o__Herpetosiphonales;f__;g__	0.9%	1.1%	0.2%	2.2%	0.0%
k__Bacteria;p__Chloroflexi;c__Chloroflexi;o__[Roseiflexales];f__;g__	0.0%	0.0%	0.0%	0.1%	0.0%
k__Bacteria;p__Chloroflexi;c__TK17;o__mle1-48;f__;g__	0.0%	0.0%	0.0%	0.1%	0.0%
k__Bacteria;p__Cyanobacteria;c__4C0d-2;o__MLE1-12;f__;g__	0.1%	0.1%	0.1%	0.0%	0.2%
k__Bacteria;p__Cyanobacteria;c__Chloroplast;o__Stramenopiles;f__;g__	1.2%	0.8%	2.2%	2.0%	0.0%
k__Bacteria;p__Cyanobacteria;c__ML635J-21;o__;f__;g__	0.3%	0.6%	0.5%	0.0%	0.0%
k__Bacteria;p__Cyanobacteria;c__Oscillatoriophycideae;o__Chroococcales;f__Spirulinaceae;g__Spirulina	0.1%	0.1%	0.3%	0.1%	0.0%
k__Bacteria;p__Cyanobacteria;c__Oscillatoriophycideae;o__Chroococcales;f__Xenococcaceae;g__	0.0%	0.0%	0.0%	0.2%	0.0%
k__Bacteria;p__Cyanobacteria;c__Oscillatoriophycideae;o__Oscillatoriales;f__Phormidiaceae;g__	0.7%	0.0%	0.0%	2.8%	0.0%
k__Bacteria;p__Cyanobacteria;c__Oscillatoriophycideae;o__Oscillatoriales;f__Phormidiaceae;g__Phormidium	0.2%	0.0%	0.8%	0.0%	0.0%
k__Bacteria;p__Cyanobacteria;c__Synechococcophycideae;o__Pseudanabaenales;f__Pseudanabaenaceae;g__Leptolyngbya	0.1%	0.0%	0.0%	0.2%	0.0%
k__Bacteria;p__Elusimicrobia;c__Elusimicrobia;o__Elusimicrobiales;f__;g__	0.1%	0.2%	0.1%	0.0%	0.0%
k__Bacteria;p__Firmicutes;c__Clostridia;o__Clostridiales;f__Lachnospiraceae;g__	0.2%	0.0%	0.7%	0.0%	0.0%
k__Bacteria;p__Firmicutes;c__Clostridia;o__Clostridiales;f__[Acidaminobacteraceae];g__Acidaminobacter	0.1%	0.0%	0.3%	0.0%	0.0%
k__Bacteria;p__Firmicutes;c__Clostridia;o__Clostridiales;f__[Acidaminobacteraceae];g__Fusibacter	0.3%	0.0%	1.3%	0.0%	0.0%
k__Bacteria;p__Firmicutes;c__Erysipelotrichi;o__Erysipelotrichales;f__Erysipelotrichaceae;g__PSB-M-3	0.2%	0.0%	0.7%	0.0%	0.0%
k__Bacteria;p__Fusobacteria;c__Fusobacteriia;o__Fusobacteriales;f__;g__	0.4%	0.1%	1.5%	0.0%	0.0%
k__Bacteria;p__GN02;c__;o__;f__;g__	0.0%	0.1%	0.1%	0.0%	0.0%
k__Bacteria;p__GN02;c__BD1-5;o__;f__;g__	0.2%	0.2%	0.5%	0.0%	0.0%
k__Bacteria;p__GN04;c__GN15;o__;f__;g__	0.1%	0.0%	0.1%	0.2%	0.0%
k__Bacteria;p__GN04;c__MSB-5A5;o__;f__;g__	0.0%	0.0%	0.0%	0.2%	0.0%
k__Bacteria;p__Gemmatimonadetes;c__Gemm-1;o__;f__;g__	0.1%	0.1%	0.2%	0.0%	0.0%
k__Bacteria;p__NKB19;c__;o__;f__;g__	0.1%	0.1%	0.1%	0.2%	0.2%
k__Bacteria;p__Nitrospirae;c__Nitrospira;o__Nitrospirales;f__;g__	0.1%	0.1%	0.2%	0.0%	0.0%
k__Bacteria;p__Nitrospirae;c__Nitrospira;o__Nitrospirales;f__Nitrospiraceae;g__	0.0%	0.1%	0.0%	0.1%	0.0%
k__Bacteria;p__Nitrospirae;c__Nitrospira;o__Nitrospirales;f__Nitrospiraceae;g__Nitrospira	15.7%	7.4%	5.6%	24.5%	25.4 %
k__Bacteria;p__Nitrospirae;c__Nitrospira;o__Nitrospirales;f__[Thermodesulfovibrionaceae];g__GOUTA19	0.1%	0.1%	0.0%	0.4%	0.0%
k__Bacteria;p__OD1;c__;o__;f__;g__	0.1%	0.0%	0.0%	0.2%	0.0%
k__Bacteria;p__OP3;c__PBS-25;o__;f__;g__	0.0%	0.1%	0.1%	0.0%	0.0%
k__Bacteria;p__Planctomycetes;c__OM190;o__CL500-15;f__;g__	0.1%	0.2%	0.1%	0.0%	0.0%
k__Bacteria;p__Planctomycetes;c__OM190;o__agg27;f__;g__	0.2%	0.1%	0.0%	0.7%	0.0%
k__Bacteria;p__Planctomycetes;c__Phycisphaerae;o__CCM11a;f__;g__	0.5%	0.7%	0.5%	0.8%	0.0%
k__Bacteria;p__Planctomycetes;c__Phycisphaerae;o__Phycisphaerales;f__;g__	0.2%	0.3%	0.2%	0.0%	0.3%
k__Bacteria;p__Planctomycetes;c__Phycisphaerae;o__Phycisphaerales;f__Phycisphaeraceae;g__	0.1%	0.1%	0.2%	0.0%	0.0%
k__Bacteria;p__Planctomycetes;c__Phycisphaerae;o__WD2101;f__;g__	0.1%	0.0%	0.2%	0.1%	0.0%
k__Bacteria;p__Planctomycetes;c__Phycisphaerae;o__mle1-8;f__;g__	0.1%	0.0%	0.1%	0.1%	0.0%
k__Bacteria;p__Planctomycetes;c__Planctomycetia;o__Gemmatales;f__Gemmataceae;g__	0.2%	0.0%	0.0%	0.7%	0.0%

k__Bacteria;p__Planctomycetes;c__Planctomycetia;o__Gemmatales;f__Gemmataceae;g__Gemmata	0.0%	0.1%	0.0%	0.1%	0.0%
k__Bacteria;p__Planctomycetes;c__Planctomycetia;o__Pirellulales;f__Pirellulaceae;g__	0.5%	0.1%	0.2%	0.7%	1.1%
k__Bacteria;p__Planctomycetes;c__Planctomycetia;o__Pirellulales;f__Pirellulaceae;g__A17	0.1%	0.0%	0.0%	0.3%	0.2%
k__Bacteria;p__Planctomycetes;c__Planctomycetia;o__Pirellulales;f__Pirellulaceae;g__Pirellula	0.2%	0.1%	0.0%	0.8%	0.0%
k__Bacteria;p__Planctomycetes;c__Planctomycetia;o__Planctomycetales;f__Planctomycetaceae;g__Planctomyces	0.9%	0.9%	0.8%	0.7%	1.3%
k__Bacteria;p__Planctomycetes;c__[Brocadiae];o__Brocadiales;f__W4;g__	0.0%	0.0%	0.0%	0.1%	0.0%
k__Bacteria;p__Planctomycetes;c__vadinHA49;o__DH61;f__;g__	0.1%	0.0%	0.0%	0.3%	0.0%
k__Bacteria;p__Planctomycetes;c__vadinHA49;o__p04_C01;f__;g__	0.1%	0.0%	0.2%	0.1%	0.1%
k__Bacteria;p__Proteobacteria;c__Alphaproteobacteria;o__;f__;g__	1.7%	0.4%	0.4%	0.1%	6.0%
k__Bacteria;p__Proteobacteria;c__Alphaproteobacteria;o__BD7-3;f__;g__	0.1%	0.3%	0.2%	0.0%	0.0%
k__Bacteria;p__Proteobacteria;c__Alphaproteobacteria;o__Caulobacterales;f__Caulobacteraceae;g__	0.1%	0.2%	0.1%	0.0%	0.0%
k__Bacteria;p__Proteobacteria;c__Alphaproteobacteria;o__Caulobacterales;f__Caulobacteraceae;g__Phenylobacterium	0.1%	0.1%	0.0%	0.0%	0.0%
k__Bacteria;p__Proteobacteria;c__Alphaproteobacteria;o__Rhizobiales;f__;g__	0.1%	0.0%	0.0%	0.1%	0.4%
k__Bacteria;p__Proteobacteria;c__Alphaproteobacteria;o__Rhizobiales;f__Bradyrhizobiaceae;g__	0.1%	0.0%	0.2%	0.0%	0.0%
k__Bacteria;p__Proteobacteria;c__Alphaproteobacteria;o__Rhizobiales;f__Hyphomicrobiaceae;g__	0.0%	0.0%	0.0%	0.1%	0.0%
k__Bacteria;p__Proteobacteria;c__Alphaproteobacteria;o__Rhizobiales;f__Hyphomicrobiaceae;g__Rhodoplanes	0.0%	0.0%	0.0%	0.1%	0.1%
k__Bacteria;p__Proteobacteria;c__Alphaproteobacteria;o__Rhizobiales;f__Methylocystaceae;g__Methylosinus	0.1%	0.1%	0.1%	0.4%	0.0%
k__Bacteria;p__Proteobacteria;c__Alphaproteobacteria;o__Rhizobiales;f__Rhizobiaceae;Other	0.1%	0.2%	0.1%	0.0%	0.0%
k__Bacteria;p__Proteobacteria;c__Alphaproteobacteria;o__Rhizobiales;f__Rhizobiaceae;g__	0.1%	0.5%	0.1%	0.0%	0.0%
k__Bacteria;p__Proteobacteria;c__Alphaproteobacteria;o__Rhizobiales;f__Rhizobiaceae;g__Agrobacterium	0.1%	0.0%	0.3%	0.0%	0.0%
k__Bacteria;p__Proteobacteria;c__Alphaproteobacteria;o__Rhodobacterales;f__Hyphomonadaceae;g__	2.5%	3.5%	1.7%	4.9%	0.0%
k__Bacteria;p__Proteobacteria;c__Alphaproteobacteria;o__Rhodobacterales;f__Rhodobacteraceae;g__	0.1%	0.3%	0.2%	0.0%	0.0%
k__Bacteria;p__Proteobacteria;c__Alphaproteobacteria;o__Rhodobacterales;f__Rhodobacteraceae;g__Rhodobacter	0.1%	0.1%	0.3%	0.0%	0.0%
k__Bacteria;p__Proteobacteria;c__Alphaproteobacteria;o__Rhodobacterales;f__Rhodobacteraceae;g__Rubellimicrobiu m	0.1%	0.1%	0.2%	0.0%	0.0%
k__Bacteria;p__Proteobacteria;c__Alphaproteobacteria;o__Rhodospirillales;f__Rhodospirillaceae;g__	0.9%	0.8%	0.5%	1.0%	1.4%
k__Bacteria;p__Proteobacteria;c__Alphaproteobacteria;o__Rickettsiales;f__;g__	0.3%	0.1%	0.1%	0.0%	0.8%
k__Bacteria;p__Proteobacteria;c__Alphaproteobacteria;o__Sphingomonadales;Other;Other	0.2%	0.5%	0.3%	0.0%	0.0%
k__Bacteria;p__Proteobacteria;c__Alphaproteobacteria;o__Sphingomonadales;f__;g__	0.8%	2.1%	0.9%	0.2%	0.0%
k__Bacteria;p__Proteobacteria;c__Alphaproteobacteria;o__Sphingomonadales;f__Erythrobacteraceae;g__	0.2%	0.4%	0.4%	0.0%	0.0%
k__Bacteria;p__Proteobacteria;c__Alphaproteobacteria;o__Sphingomonadales;f__Sphingomonadaceae;g__	0.5%	1.2%	0.9%	0.1%	0.0%
k__Bacteria;p__Proteobacteria;c__Alphaproteobacteria;o__Sphingomonadales;f__Sphingomonadaceae;g__Kaistobacter	0.2%	0.2%	0.7%	0.0%	0.0%
k__Bacteria;p__Proteobacteria;c__Alphaproteobacteria;o__Sphingomonadales;f__Sphingomonadaceae;g__Novosphing obium	0.1%	0.1%	0.1%	0.0%	0.0%
k__Bacteria;p__Proteobacteria;c__Alphaproteobacteria;o__Sphingomonadales;f__Sphingomonadaceae;g__Sphingomon as	0.3%	0.2%	0.0%	0.0%	0.9%
k__Bacteria;p__Proteobacteria;c__Alphaproteobacteria;o__Sphingomonadales;f__Sphingomonadaceae;g__Sphingopyxi s	0.0%	0.1%	0.1%	0.0%	0.0%

k__Bacteria;p__Proteobacteria;c__Betaproteobacteria;Other;Other;Other	2.8%	1.0%	2.6%	7.5%	0.2%
k__Bacteria;p__Proteobacteria;c__Betaproteobacteria;o__;f__;g__	5.3%	10.6%	9.5%	0.9%	0.1%
k__Bacteria;p__Proteobacteria;c__Betaproteobacteria;o__Burkholderiales;f__;g__	0.1%	0.1%	0.4%	0.0%	0.0%
k__Bacteria;p__Proteobacteria;c__Betaproteobacteria;o__Burkholderiales;f__Comamonadaceae;Other	0.3%	0.1%	0.5%	0.6%	0.1%
k__Bacteria;p__Proteobacteria;c__Betaproteobacteria;o__Burkholderiales;f__Comamonadaceae;g__	3.0%	5.1%	4.1%	2.4%	0.6%
k__Bacteria;p__Proteobacteria;c__Betaproteobacteria;o__Burkholderiales;f__Comamonadaceae;g__Acidovorax	0.2%	0.6%	0.1%	0.0%	0.0%
k__Bacteria;p__Proteobacteria;c__Betaproteobacteria;o__Burkholderiales;f__Comamonadaceae;g__Comamonas	0.1%	0.3%	0.1%	0.1%	0.0%
k__Bacteria;p__Proteobacteria;c__Betaproteobacteria;o__Burkholderiales;f__Comamonadaceae;g__Hydrogenophaga	1.4%	2.1%	1.0%	1.0%	1.3%
k__Bacteria;p__Proteobacteria;c__Betaproteobacteria;o__Burkholderiales;f__Comamonadaceae;g__Leptothrix	0.1%	0.1%	0.1%	0.0%	0.0%
k__Bacteria;p__Proteobacteria;c__Betaproteobacteria;o__Burkholderiales;f__Comamonadaceae;g__Limnobacter	0.2%	0.1%	0.1%	0.6%	0.0%
k__Bacteria;p__Proteobacteria;c__Betaproteobacteria;o__Burkholderiales;f__Comamonadaceae;g__Methylibium	0.0%	0.1%	0.0%	0.1%	0.0%
k__Bacteria;p__Proteobacteria;c__Betaproteobacteria;o__Burkholderiales;f__Comamonadaceae;g__Variovorax	0.1%	0.1%	0.2%	0.1%	0.0%
k__Bacteria;p__Proteobacteria;c__Betaproteobacteria;o__Burkholderiales;f__Oxalobacteraceae;g__	0.3%	0.9%	0.1%	0.2%	0.0%
k__Bacteria;p__Proteobacteria;c__Betaproteobacteria;o__Burkholderiales;f__Oxalobacteraceae;g__Cupriavidus	0.1%	0.3%	0.1%	0.0%	0.0%
k__Bacteria;p__Proteobacteria;c__Betaproteobacteria;o__Gallionellales;f__Gallionellaceae;g__Gallionella	5.9%	6.2%	8.9%	7.2%	1.2%
k__Bacteria;p__Proteobacteria;c__Betaproteobacteria;o__Methylophilales;f__Methylophilaceae;g__	0.1%	0.1%	0.2%	0.0%	0.0%
k__Bacteria;p__Proteobacteria;c__Betaproteobacteria;o__Neisseriales;f__Neisseriaceae;Other	0.4%	1.4%	0.1%	0.0%	0.0%
k__Bacteria;p__Proteobacteria;c__Betaproteobacteria;o__Neisseriales;f__Neisseriaceae;g__	0.4%	0.8%	0.5%	0.4%	0.0%
k__Bacteria;p__Proteobacteria;c__Betaproteobacteria;o__Neisseriales;f__Neisseriaceae;g__Chromobacterium	0.0%	0.0%	0.0%	0.0%	0.1%
k__Bacteria;p__Proteobacteria;c__Betaproteobacteria;o__Neisseriales;f__Neisseriaceae;g__Vogesella	0.2%	0.8%	0.1%	0.1%	0.0%
k__Bacteria;p__Proteobacteria;c__Betaproteobacteria;o__Nitrosomonadales;f__Nitrosomonadaceae;g__	1.9%	0.0%	0.0%	0.3%	7.4%
k__Bacteria;p__Proteobacteria;c__Betaproteobacteria;o__Procabacteriales;f__Procabacteriaceae;g__	0.1%	0.1%	0.2%	0.0%	0.0%
k__Bacteria;p__Proteobacteria;c__Betaproteobacteria;o__Rhodocyclales;f__Rhodocyclaceae;Other	0.8%	0.2%	2.9%	0.1%	0.0%
k__Bacteria;p__Proteobacteria;c__Betaproteobacteria;o__Rhodocyclales;f__Rhodocyclaceae;g__	1.2%	0.4%	2.3%	2.0%	0.0%
k__Bacteria;p__Proteobacteria;c__Betaproteobacteria;o__Rhodocyclales;f__Rhodocyclaceae;g__Azoarcus	1.3%	0.4%	4.8%	0.0%	0.0%
k__Bacteria;p__Proteobacteria;c__Betaproteobacteria;o__Rhodocyclales;f__Rhodocyclaceae;g__Dechloromonas	0.6%	0.9%	1.5%	0.0%	0.0%
k__Bacteria;p__Proteobacteria;c__Betaproteobacteria;o__Rhodocyclales;f__Rhodocyclaceae;g__Sulfuritalea	0.1%	0.1%	0.3%	0.0%	0.0%
k__ Bacteria;p__ Proteobacteria;c__ Betaproteobacteria;o__SBla14;f__;g__	1.2%	1.0%	2.6%	1.2%	0.0%
k__Bacteria;p__Proteobacteria;c__Betaproteobacteria;o__Thiobacterales;f__;g__	0.2%	0.3%	0.2%	0.3%	0.0%
k__Bacteria;p__Proteobacteria;c__Deltaproteobacteria;o__Bdellovibrionales;f__Bacteriovoracaceae;g__	0.8%	0.4%	0.8%	0.6%	1.2%
k__Bacteria;p__Proteobacteria;c__Deltaproteobacteria;o__Bdellovibrionales;f__Bdellovibrionaceae;g__Bdellovibrio	2.4%	0.5%	0.3%	0.4%	8.4%
k__Bacteria;p__Proteobacteria;c__Deltaproteobacteria;o__Desulfuromonadales;Other;Other	0.6%	0.0%	0.8%	1.6%	0.0%
k__Bacteria;p__Proteobacteria;c__Deltaproteobacteria;o__Desulfuromonadales;f__Desulfuromonadaceae;g__	0.1%	0.0%	0.3%	0.0%	0.0%
k__Bacteria;p__Proteobacteria;c__Deltaproteobacteria;o__Desulfuromonadales;f__Geobacteraceae;g__Geobacter	1.3%	0.1%	3.6%	1.5%	0.0%
k__Bacteria;p__Proteobacteria;c__Deltaproteobacteria;o__Desulfuromonadales;f__Pelobacteraceae;g__	0.1%	0.0%	0.5%	0.0%	0.0%
k__Bacteria;p__Proteobacteria;c__Deltaproteobacteria;o__Myxococcales;f__;g__	0.3%	0.8%	0.1%	0.3%	0.0%
k__Bacteria;p__Proteobacteria;c__Deltaproteobacteria;o__Myxococcales;f__0319-6G20;g__	0.1%	0.0%	0.0%	0.0%	0.3%
k__Bacteria;p__Proteobacteria;c__Deltaproteobacteria;o__Myxococcales;f__Haliangiaceae;g__	0.0%	0.1%	0.1%	0.0%	0.0%

k__Bacteria;p__Proteobacteria;c__Deltaproteobacteria;o__Myxococcales;f__OM27;g__	0.0%	0.1%	0.0%	0.1%	0.0%
k__Bacteria;p__Proteobacteria;c__Deltaproteobacteria;o__Spirobacillales;f__;g__	0.2%	0.1%	0.4%	0.0%	0.0%
k__Bacteria;p__Proteobacteria;c__Deltaproteobacteria;o__Syntrophobacterales;f__Syntrophobacteraceae;g__	0.1%	0.1%	0.1%	0.2%	0.0%
k__Bacteria;p__Proteobacteria;c__Epsilonproteobacteria;o__Campylobacterales;f__Campylobacteraceae;g__Arcobacter	0.5%	1.5%	0.4%	0. 0%	0.0%
k__Bacteria;p__Proteobacteria;c__Epsilonproteobacteria;o__Campylobacterales;f__Helicobacteraceae;g__Sulfuricurvu m	0.0%	0.0%	0.0%	0.1%	0.0%
k__Bacteria;p__Proteobacteria;c__Gammaproteobacteria;o__;f__;g__	0.1%	0.2%	0.1%	0.0%	0.0%
k__Bacteria;p__Proteobacteria;c__Gammaproteobacteria;o__34P16;f__;g__	0.2%	0.5%	0.4%	0.0%	0.1%
k__Bacteria;p__Proteobacteria;c__Gammaproteobacteria;o__Aeromonadales;f__Aeromonadaceae;g__	0.4%	0.9%	0.6%	0.2%	0.0%
k__Bacteria;p__Proteobacteria;c__Gammaproteobacteria;o__Alteromonadales;f__;g__	0.1%	0.1%	0.1%	0.0%	0.0%
k__Bacteria;p__Proteobacteria;c__Gammaproteobacteria;o__Alteromonadales;f__Alteromonadaceae;g__	0.1%	0.3%	0.1%	0.0%	0.0%
k__Bacteria;p__Proteobacteria;c__Gammaproteobacteria;o__Alteromonadales;f__Alteromonadaceae;g__Cellvibrio	1.9%	5.6%	1.2%	0.6%	0.3%
k__Bacteria;p__Proteobacteria;c__Gammaproteobacteria;o__Alteromonadales;f__HTCC2188;g__HTCC	0.5%	0.1%	0.0%	1.6%	0.4%
k__Bacteria;p__Proteobacteria;c__Gammaproteobacteria;o__Alteromonadales;f__Shewanellaceae;g__Shewanella	0.1%	0.1%	0.2%	0.0%	0.0%
k__Bacteria;p__Proteobacteria;c__Gammaproteobacteria;o__Alteromonadales;f__[Chromatiaceae];g__Rheinheimera	1.9%	4.7%	1.7%	1.2%	0.1%
k__Bacteria;p__Proteobacteria;c__Gammaproteobacteria;o__Enterobacteriales;f__Enterobacteriaceae;g__Erwinia	0.1%	0.1%	0.2%	0.0%	0.0%
k__Bacteria;p__Proteobacteria;c__Gammaproteobacteria;o__HTCC2188;f__;g__	0.0%	0.0%	0.0%	0.1%	0.1%
k__Bacteria;p__Proteobacteria;c__Gammaproteobacteria;o__Legionellales;f__Coxiellaceae;g__	0.1%	0.0%	0.0%	0.0%	0.2%
k__Bacteria;p__Proteobacteria;c__Gammaproteobacteria;o__Methylococcales;Other;Other	1.0%	2.1%	1.5%	0.5%	0.0%
k__Bacteria;p__Proteobacteria;c__Gammaproteobacteria;o__Methylococcales;f__Crenotrichaceae;g__Crenothrix	0.9%	0.5%	0.4%	1.0%	1.9%
k__Bacteria;p__Proteobacteria;c__Gammaproteobacteria;o__Methylococcales;f__Methylococcaceae;g__Methylomonas	0.1%	0.2%	0.1%	0.0%	0.0%
k__Bacteria;p__Proteobacteria;c__Gammaproteobacteria;o__Oceanospirillales;f__Oleiphilaceae;g__	0.3%	0.8%	0.2%	0.0%	0.0%
k__Bacteria;p__Proteobacteria;c__Gammaproteobacteria;o__PYR10d3;f__;g__	0.2%	0.0%	0.0%	0.0%	0.6%
k__Bacteria;p__Proteobacteria;c__Gammaproteobacteria;o__Pseudomonadales;f__Moraxellaceae;g__	0.4%	0.2%	0.1%	1.4%	0.0%
k__Bacteria;p__Proteobacteria;c__Gammaproteobacteria;o__Pseudomonadales;f__Moraxellaceae;g__Acinetobacter	0.2%	0.8%	0.1%	0.0%	0.0%
k__Bacteria;p__Proteobacteria;c__Gammaproteobacteria;o__Pseudomonadales;f__Moraxellaceae;g__Perlucidibaca	0.6%	0.1%	0.0%	0.9%	1.2%
k__Bacteria;p__Proteobacteria;c__Gammaproteobacteria;o__Pseudomonadales;f__Pseudomonadaceae;g__	0.3%	0.9%	0.3%	0.0%	0.0%
k__Bacteria;p__Proteobacteria;c__Gammaproteobacteria;o__Pseudomonadales;f__Pseudomonadaceae;g__Pseudomona s	2.0%	5.9%	1.6%	0.3%	0.3%
k__Bacteria;p__Proteobacteria;c__Gammaproteobacteria;o__Thiotrichales;f__Piscirickettsiaceae;g__Methylophaga	0.3%	0.8%	0.3%	0.0%	0.0%
k__Bacteria;p__Proteobacteria;c__Gammaproteobacteria;o__Xanthomonadales;f__Sinobacteraceae;g__	0.4%	0.3%	0.2%	1.3%	0.0%
k__Bacteria;p__Proteobacteria;c__Gammaproteobacteria;o__Xanthomonadales;f__Xanthomonadaceae;g__	0.2%	0.0%	0.0%	0.4%	0.3%
k__Bacteria;p__Proteobacteria;c__Zetaproteobacteria;o__Mariprofundales;f__Mariprofundaceae;g__Mariprofundus	0.1%	0.0%	0.0%	0.5%	0.0%
k__Bacteria;p__SBR1093;c__VHS-B5-50;o__;f__;g__	0.1%	0.0%	0.0%	0.1%	0.0%
k__Bacteria;p__TM7;c__TM7-1;o__;f__;g__	0.1%	0.0%	0.0%	0.2%	0.0%
k__Bacteria;p__Verrucomicrobia;c__Opitutae;o__Opitutales;f__Opitutaceae;g__	6.9%	0.8%	2.4%	0.5%	24.0 %
k__Bacteria;p__Verrucomicrobia;c__Verrucomicrobiae;o__Verrucomicrobiales;f__Verrucomicrobiaceae;g__	0.0%	0.1%	0.1%	0.0%	0.0%

k__Bacteria;p__Verrucomicrobia;c__[Pedosphaerae];o__;f__;g__	0.0%	0.0%	0.1%	0.0%	0.0%
k__Bacteria;p__Verrucomicrobia;c__[Pedosphaerae];o__[Pedosphaerales];f__;g__	0.1%	0.1%	0.2%	0.2%	0.1%
k__Bacteria;p__Verrucomicrobia;c__[Pedosphaerae];o__[Pedosphaerales];f__R4-41B;g__	0.1%	0.1%	0.0%	0.0%	0.0%
k__Bacteria;p__Verrucomicrobia;c__[Pedosphaerae];o__[Pedosphaerales];f__auto67_4W;g__	0.3%	0.2%	0.7%	0.1%	0.0%
k__Bacteria;p__WS3;c__PRR-12;o__;f__;g__	0.1%	0.0%	0.1%	0.1%	0.0%

Supplementary Table 4. Number of bacteria isolated from LT and VO in different selective media containing Mn(II).

Growth Media	Number of Isolates	
	LT	VO
PC Medium	54	34
Mn Medium	3	40
Lept Medium	43	28
Total	100	102
